# Genetics of constant and severe pain in the NAPS2 cohort of recurrent acute and chronic pancreatitis patients^☆^

**DOI:** 10.1016/j.jpain.2024.104754

**Published:** 2024-12-12

**Authors:** Ellyn K. Dunbar, Phil J. Greer, Jami L. Saloman, Kathryn M. Albers, Dhiraj Yadav, David C. Whitcomb

**Affiliations:** a Department of Medicine, University of Pittsburgh, Pittsburgh, PA, USA; b Department of Human Genetics, Graduate School of Public Health, University of Pittsburgh, Pittsburgh, PA, USA; c Department of Neurobiology, Pittsburgh Center for Pain Research, University of Pittsburgh, Pittsburgh, PA, USA; d Pittsburgh Center for Pain Research, University of Pittsburgh, Pittsburgh, PA, USA; e Department of Cell Biology & Molecular Physiology, University of Pittsburgh, Pittsburgh, PA, USA

**Keywords:** Pancreatitis, Chronic pain, Neuralgia, Genetics, Inflammation

## Abstract

Recurrent acute and chronic pancreatitis (RAP, CP) are complex, progressive inflammatory diseases with variable pain experiences impacting patient function and quality of life. The genetic variants and pain pathways in patients contributing to most severe pain experiences are unknown. We used previously genotyped individuals with RAP/CP from the North American Pancreatitis Study II (NAPS2) of European Ancestry for nested genome-wide associated study (GWAS) for pain-severity, chronicity, or both. Lead variants from GWAS were determined using FUMA. Loci with p<1e-5 were identified for *post-hoc* candidate identification. Transcriptome-wide association studies (TWAS) identified loci in *cis* and *trans* to the lead variants. Serum from phenotyped individuals with CP from the PROspective Evaluation of Chronic Pancreatitis for EpidEmiologic and Translational StuDies (PROCEED) was assessed for BDNF levels using Meso Scale Discovery Immunoassay. We identified four pain systems defined by candidate genes: 1) Pancreas-associated injury/stress mitigation genes include: *REG* gene cluster, *CTRC*, *NEURL3* and *HSF2*2. 2) Neural development and axon guidance tracing genes include: *SNPO, RGMA, MAML1* and *DOK6* (part of the *RET* complex). 3) Genes linked to psychiatric stress disorders include *TMEM65*, *RBFOX1,* and *ZNF385D.* 4) Genes in the dorsal horn pain-modulating BDNF/neuropathic pathway included *SYNPR*, *NTF3 and RBFOX1*. In an independent cohort BDNF was significantly elevated in patients with *constantsevere pain*. Extension and expansion of this exploratory study may identify pathway- and mechanism-dependent targets for individualized pain treatments in CP patients.

**Perspective:**

Pain is the most distressing and debilitating feature of chronic pancreatitis. Yet many patients with chronic pancreatitis have little or no pain. The North American Pancreatitis Study II (NAPS2) includes over 1250 pancreatitis patients of all progressive stages with all clinical and phenotypic characteristics carefully recorded. Pain did not correlate well with disease stage, inflammation, fibrosis or other features. Here we spit the patients into groups with the most severe pain and/or chronic pain syndromes and compared them genetically with patients reporting mild or minimal pain. Although some genetic variants associated with pain were expressed in cells (1) of the pancreas, most genetic variants were linked to genes expressed in the nervous system cells associated with (2) neural development and axon guidance (as needed for the descending inhibition pathway), (3) psychiatric stress disorders, and (4) cells regulating sensory nerves associated with BDNF and neuropathic pain. Similar and overlapping genetic variants in systems 2 – 4 are also seen in pain syndromes form other organs. The implications for treating pancreatic pain are great in that we can no longer focus on just the pancreas. Furthermore, new treatments designed for pain disorders in other tissues may be effective in some patient with pain syndromes from the pancreas. Further research is needed to replicate and extend these observations so that new, genetics-guided rational treatments can be developed and delivered.

## Introduction

Pancreatitis is an inflammatory disorder of the pancreas with many etiologies that may irreversibly damage the tissue leading to organ dysfunction or failure.^1–4^ Pancreatitis often starts with episodes of acute pancreatitis (AP) that variably progresses to recurrent acute pancreatitis (RAP) and finally chronic pancreatitis (CP).^1–3,5,6^ All stages of disease are accompanied by diminished physical and mental quality of life (QOL) and the major driver of these debilitating outcomes is pain.^7–9^

The most debilitating symptom of pancreatitis is severe, constant pain, which is seen in 1 out of 3 CP patients.^1,7–10^ Pancreatitis pain is especially challenging to manage as treatments using medications, endoscopy or surgery are often ineffective.^11,12^ Additionally, the pain experienced by RAP and CP patients varies in frequency, character, severity and chronicity even within patients with similar disease states.^1, 11–14^ Furthermore, pancreatitis pain does not correlate with abdominal imaging, exocrine pancreatic insufficiency (EPI) or other common features, suggesting additional regulation by genetic and environmental risks affecting the immune system, the nervous system, psychosocial systems or complex combinations of multiple factors in multiple systems.^15–18^ Pain modulating systems that may be affected by genetic variants that are involved in pancreatitis-associated pain syndromes include local inflammation and neural systems including GABAergic, catecholaminergic, cytokines, growth factors, serotonergic, estrogenic, glutamatergic, proteinases, axonogenesis, nervous-system development, and neural connectivity.^19–21^ These have not been systematically assessed as part of pancreatic pain syndromes.

Pain and suffering are complex concepts that are difficult to measure and phenotype because both sensory and emotional components contribute to the individual patient’s experience. Although pain and poor QOL in pancreatitis are the most important clinical consideration for patients, insights into the underlying mechanisms of pancreatitis pain have been difficult to study beyond endoscopic or surgical drainage procedures for obstructed pancreatic ducts or surgical resections or total pancreatectomy with islet autotransplantation (TPIAT). More systematic approaches include new patient reported outcomes (PRO) such as the COMPAT-SF ^22^ and quantitative sensory testing (QST) ^23,24^, but patient perception and sensory testing alone do not adequately address the underlying molecular mechanisms of aberrant pain responses. Pathologic pain responses may have genetic variants that alter cellular mechanisms regulating normal pain biology.

Challenges to determining the effects of genetic variants on pain severity, quality, persistence and stress-associated psychiatric disorders include the availability of well phenotyped and genotyped patients, and inclusion of adequate PRO, endophenotype, and QST. Furthermore, most pain syndromes are not familial (i.e. Mendelian inheritance of monogenic pathogenic variants), but rather genetically complex with multiple factors contributing to dysfunctional systems. Additional complexity in phenotyping is the requirement of proximal and substantial injuries that may reveal defective pain modulating and protective systems, including stress-associated diathesis such as stress-associated psychiatric disorders.

Here we demonstrate the identification of multiple genetic risk variant-associated pain pathways for severe and/or continuous pancreatitis pain using a biological systems-based, reverse engineering approach to identify candidate pain variants known to be linked to pain and enriched in pancreatitis pain patients. These systems include (1) genetic variants that increase local inflammation; (2) variants predicted to potentiate Brain Derived Neurotrophic Factor (BDNF)-associated pain mechanisms; (3) variants that likely alter nervous-system development and connectivity (e.g. affecting the connection of inhibitory nerves to excitatory pathways); (4) variants associated with stress-associated psychiatric conditions and others.

## Methods

### Conceptual framework and approach

We propose that common pain experience-modulating genetic variants can be identified in pancreatitis patient using nested GWAS and TWAS data based on statistical logic of complex disorders and Bayesian variant selection arguments. First, if multiple genetic variants are necessary to predispose to a common condition, then the majority of these variants must be common, or they would seldom occur together. For example, if susceptibility to a condition were present in 6% of the population and 3 genetic variants were required, the minor allele frequency (MAF) of the variants would, on average, be 0.40 (0.4^3^ = 0.064). And if there were multiple susceptibility conditions with a chronic and/or severe pain phenotype, then additional genetic variants would be common in the general population.

Secondly, for complex disorders the damaging effects of some component genetic variants may be large, but the effect size masked by the conditional requirements of phenotypic expression. Thus, the apparent effect size based on case-control studies (e.g. GWAS LOD score) would be statistically small while the effect on gene expression or function may be mechanistically large.

Third, implications of lowering the threshold LOD score for detecting complex risk variants is to increase false discovery rates. However, if the study aim was to replicate known variants associated with pain, then loci with marginal LOD score of 4 (e.g. ~p<0.0001) that contained known pain-associated genetic variants could be selected as candidates for pancreatitis-associated pain genes while loci without known pain genes could be rejected. In many cases this represents replication of established loci rather than discovery, further justifying a lower LOD score threshold.

Fourth, we hypothesized that many of the pain syndrome-associated genetic variants are conditional in phenotypic expression, so we chose to limit analysis to patients who all had the same proximal severe injury of RAP or CP. We further hypothesized that the specific, restricted location of the affected body part (i.e. the pancreas) and the type of injury (i.e. acute injury with innate immune responses of acute and chronic inflammation) would limit the number and type of potential pathways for the pancreas. Identified pathways may potentially overlap with other pain syndromes that have been studied in genetic studies with shared central dysfunctions.

Finally, if the biological function of the gene products of the candidate genes and the type of predicted damage is known (e.g. reduced expression or function) and if they are integrated into the specialized cells and systems where they are expressed, then the specific mechanisms of pathogenic pain syndromes can be modeled, with potential treatments that may specifically overcome the genetic deficit.

The Bayesian argument of whether combinations of genetic variants in one pathway within a complex disorder (i.e. requiring more than one factor) truly identifies the pathologic system causing a stereotypic pain syndrome in a specific patient is strengthened by a lack of risk factors in alternate pathways. In the current study we chose two types of pain syndromes, increased pain severity and increased pain chronicity with the expectation that there would be both similarities and differences in genes and pathways.

We previously utilized a candidate gene method for genes associated with major depressive disorder (MDD), generalized anxiety disorder (GAD), and post-traumatic stress disorder (PTSD) with less stringent significance thresholds since only small portions of the genome were being analyzed compared to genome-wide association studies (GWAS).^13,14^ For example, threshold association levels for MDD genes overlapping with pancreatitis constant-severe pain was set at p<0.0001^13^, and for a formal analysis of GAD and PTSD candidate gene loci being associated with severe, constant-severe and constant pain loci was p<0.002 based on the number of genes/loci preselected as candidates.^14^

We also hypothesized that a genomic approach including transcriptome-wide association studies (TWAS) and expression quantitative trait loci (eQTL)-tissue colocalization could be used to leverage additional information about altered pain systems biology rather than relying only on the agnostic and study size-dependent statistical methods of GWAS.

### Patient data

#### Cohorts

The North American Pancreatitis Study II (NAPS2) cohorts served as the primary data source. Approval by the Institutional Review Board of each participating institution was granted and informed consent was obtained from each subject. These cohorts included three consecutive cross-sectional, case-control studies of individuals with RAP, CP, and phenotyped controls.^15,25,26^ Phenotypes were recorded with standardized questionnaires and DNA was genotyped using Illumina Human-OmniExpress BeadChip and HumanCoreExome.^3,13,27^ The McCarthy Group pre-imputation checking tools were used to prepare data for imputation against the 1000 genomes phase-3 reference panel on the Sanger imputation server using the EAGLE2+PBWT pipeline.^13, 28–30^ Imputation was therefore used to impute missing genotypes. The genotypes were mapped on genome build GRCh37/hg19. Patients with RAP or CP of European Ancestry (EA) were analyzed as the initial genotyping array included variants from European Ancestry cohorts.^13, 14^ Demographic data for patients are in Table 1 and Supplemental Tables S1, S2, and S3. These and other tables in this paper were generated using R (version 4.0.4) and the flextable package version 0.7.0.^31,32^

#### Pain categories

Pancreatitis pain patterns in the year prior to recruitment were described using Mullady’s 6 severity-frequency patterns where O = no pain; A = episodes of mild pain; B = constant mild to moderate pain; C = episodes of severe pain; D = constant mild and episodes of severe pain; E = constant severe pain.^1^ As done previously, individuals responding with B, D, or E were categorized as *constant pain*, individuals responding with C, D, or E were categorized as *severe pain*, and individuals responding with D or E were *constant-severe pain*.^11,13,14^ Sample sizes are reported in Table 1.

### Statistical methods

#### GWAS

Genome-wide association studies (GWAS) were performed using Plink 1.9.^33^ Since these are case-control studies, the data were fit to a logistic regression to test for associations. Covariates included age, sex, body mass index (BMI), principal components of ancestry 1–4, and a variable to control for differences across SNP chips. The threshold for minor allele frequency (MAF) was set to 0.01 and calculated with Plink 1.9 leaving 7745,456 SNPs in the analysis.^33^ The standard genome-wide levels of significance of 5×10^−8^ and suggestive significance of 1×10^−5^ were applied. Manhattan and QQ plots were generated in R (version 3.6.0) using the ggfastman package version 1.2.^34,35^

#### FUMA

Functional Mapping and Annotation (FUMA) of GWAS data from Plink was done using FUMA online.^36^ The original GWAS results based on genome build hg19 were used for compatibility with FUMA. FUMA uses linkage disequilibrium (LD) at r^2^ ≥ 0.6 to identify candidate lead SNPs within candidate genomic loci. The reference panel used to calculate LD was 1000 Genomes Project Phase 3 European. Independent lead SNPs—and their genomic loci—with a p-value less than 1×10^−5^ are reported (Tables 2, 3, 4; Supplemental Tables S4, S5, S6).

#### TWAS

Transcriptome-wide association studies (TWAS) is a post-GWAS method that uses eQTLs to identify the genes that are predicted to have differential expression associated with the phenotype.^37^ Unlike GWAS which only captures information about *cis* loci, TWAS can capture *trans* effects and biological information of loci.^38^

**Fig. 1.**
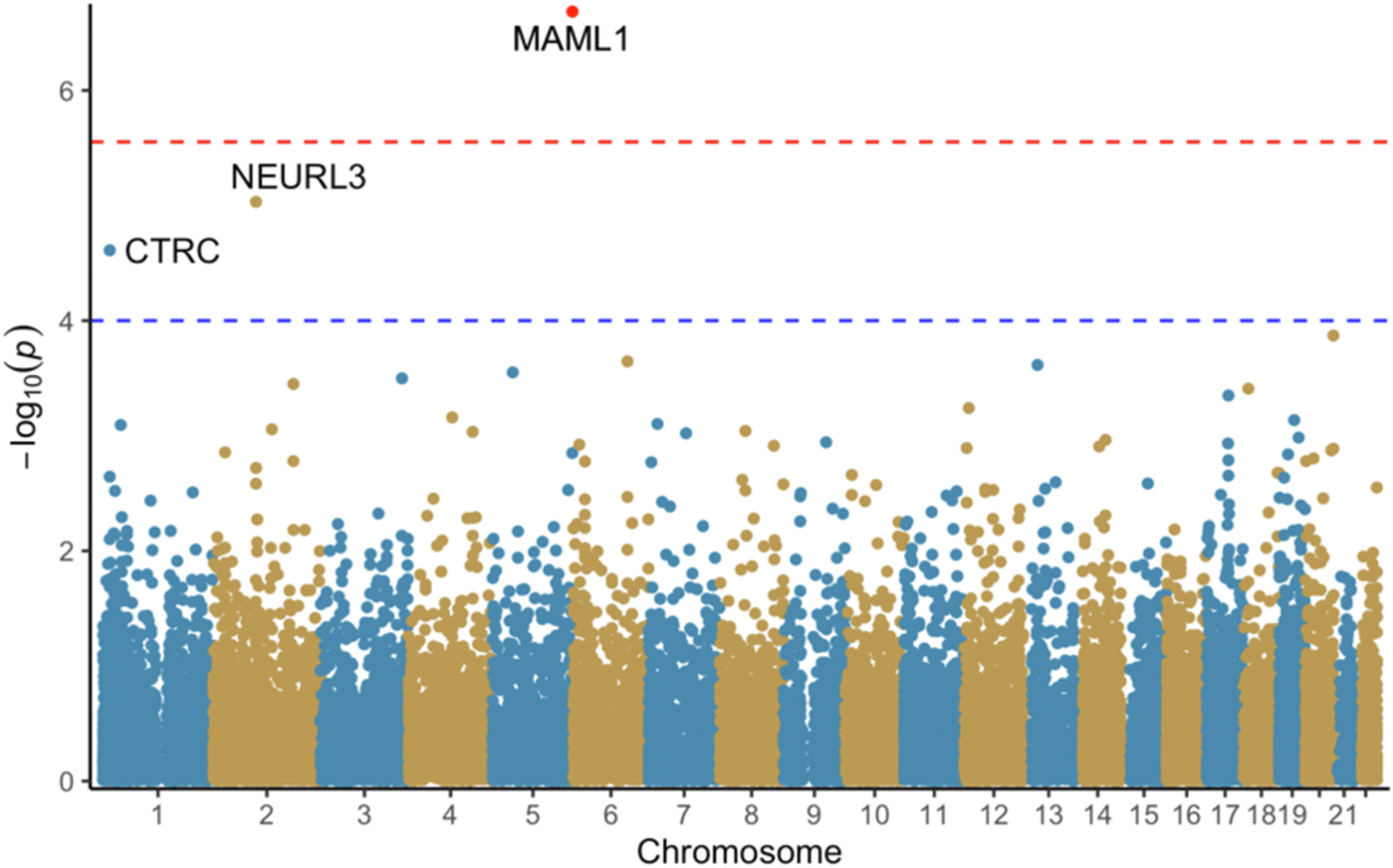
S-MultiXcan results for constant pain. Red line: p=2.8e-06. Blue line: p=1.0e-04.

**Fig. 2.**
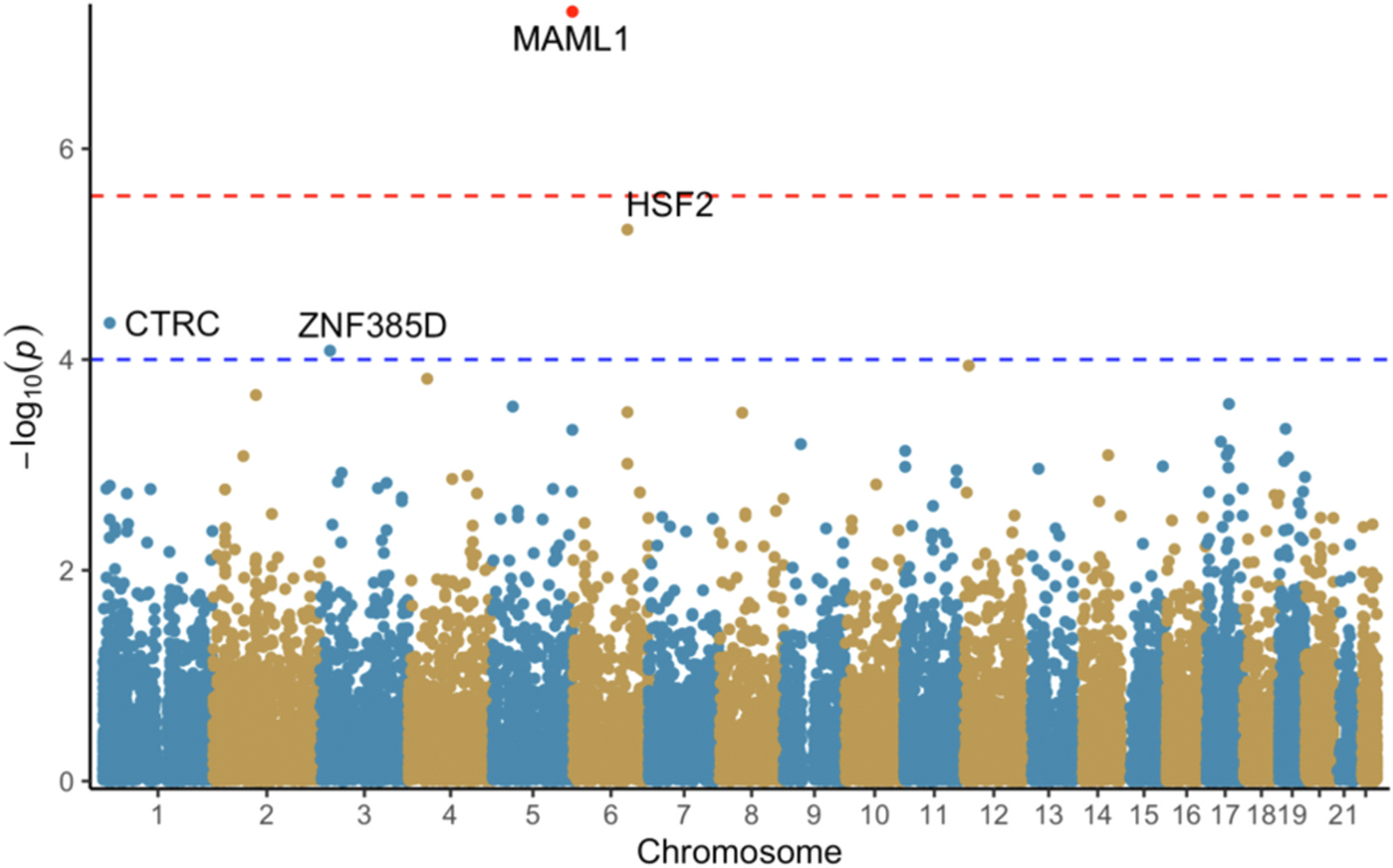
S-MultiXcan results for constant-severe pain. Red line: p=2.8e-06. Blue line: p=1.0e-04.

**Fig. 3.**
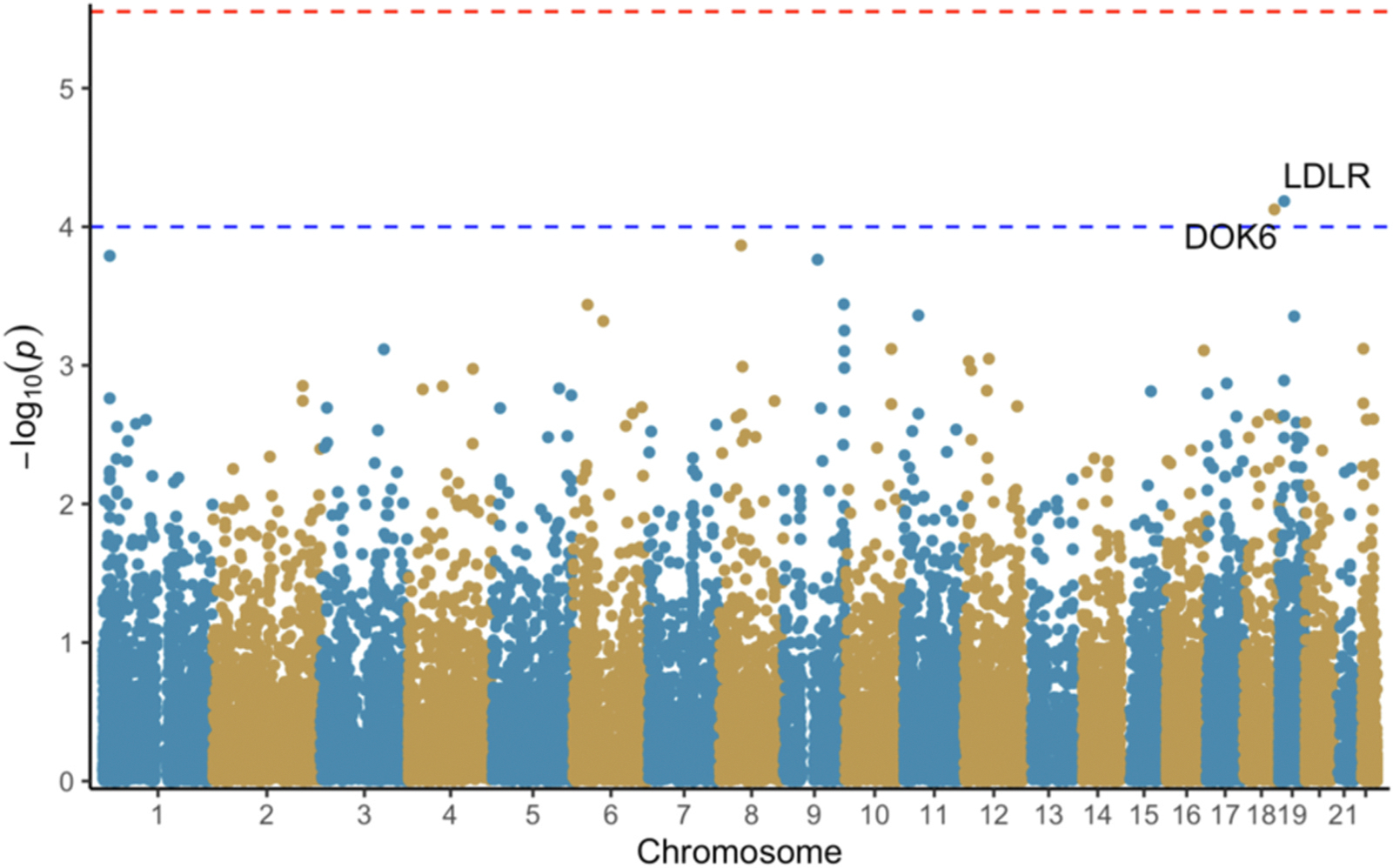
S-MultiXcan results for severe pain. Red line: p=2.8e-06. Blue line: p=1.0e-04.

The TWAS was conducted using the MetaXcan family of tools (see https://github.com/hakyimlab/MetaXcan).^39^ Auxiliary files necessary for the TWAS were downloaded from Zendo.^40^ The calculations were conducted in a Python environment provided by the authors of MetaXcan. We used methods described in the GitHub Wiki to perform full harmonization with liftover to build hg38 from build hg19, imputation of summary statistics with GTEx-v8, S-PrediXcan on the provided 49 tissues using MetaMany, and finally S-MultiXcan to produce aggregated TWAS results across all tested tissues. All available tissues were used as pain-related receptors can be expressed in all tissues.^41^ The summary versions of the calculations were used as we used summary statistics from our GWAS. MASHR-M prediction models were used as these models include more biological information than prior models.^39,42^ The significance level of 2.8×10^−6^ used for the final step of the TWAS (S-MultiXcan) was Bonferroni corrected based on the tissue with the highest number of genes tested (Testis, 17,867 genes) following Barbeira et al.^39^ Associations with a p-value smaller than 1×10^−4^ were considered suggestive significant due to LD misspecification from using summary statistics and comparing to a reference set that may not match perfectly.^39^ Graphical representations of results (Figs. 1, 2, and 3) were generated in R (version 3.6.0) using the ggfastman package version 1.2.^34,35^

### Colocalization

Colocalization is a statistical method used to determine if a disease-associated phenotype and expression phenotype are due to the same SNP(s) within a locus.^43^ We used Coloc for colocalization^43^ following instructions on the GitHub Wiki (https://chr1swallace.github.io/colo-c/index.html).Thecolocalization was conducted in R (version 3.6.0) using the coloc version 5.1.1 package.^34,43^ Coloc was performed on the significant gene-tissue pairs from S-MultiXcan. GTEx-v8 data downloaded from the GTEx Portal was used, specifically GTEx_Analysis_v8_eQTL_EUR.tar (https://storage.googleapis.com/gtex_analysis_v8/single_tissue_qtl_data/GTEx_Analysis_v8_eQTL_EUR.tar). These data included eQTLs per tissue using European Ancestry samples, which matches the ancestry of the NAPS2 data used here and the data used for the MASHR-M models from the TWAS.^42^ We used sample sizes per tissue reported in the GTEx Consortium report (Supplementary Table8).^44^ Samples sizes for our GWAS are reported in Table 1. The GWAS data formatted for the TWAS were used as it matches naming conventions of the GTEx data and contains all the SNPs included in the TWAS. SNPS from the GWAS were annotated to genes using Gencode v26, and SNPs within 1 Mbp up and downstream of the gene were included.^45,46^ The function “coloc.abf” was used to conduct the colocalization using default priors.

Coloc tests five hypotheses at any given locus using an Approximate Bayes Factor: 0) the null of no association with either trait (GWAS association signal and eQTL), 1) association with GWAS only, 2) association with eQTL only, 3) association with both traits in two independent SNPs, and 4) association with both traits in one shared SNP.^47^ The coloc procedure produces posterior probabilities (PP) for each hypothesis, with the larger probability, closer to 1, lending more support for the hypothesis.^47^ Significant evidence of colocalization was considered as a PP.H4 > 0.5, PP.H3 < 0.5, and PP.H0+PP.H1+PP.H3 < 0.3.^47^ Significant colocalizations were visualized using locuscompare in R (version 3.6.0).^34,48^

### Biomarkers

#### Serum BDNF

Pain data and serum from individuals with CP enrolled in the PRO-spective Evaluation of Chronic Pancreatitis for EpidEmiologic and Translational StuDies (PROCEED)^49^ were used to validate BDNF as a target. Pain Frequency pattern analysis included patients with no pain (n=57), intermittent pain (n=97) and constant pain (n=203); Pain Severity patterns including patients with no pain (n=57), mild-moderate pain (n=76) and severe pain (n=224); and Pain Pattern including patients with no pain (n=57) and constant-severe pain (n-167) as previously described.^50^ Serum BDNF was measured using the Meso Scale Discovery electrochemiluminescent immunoassay per the manufacturer’s instructions.^50^

## Results

We evaluated 1254 patients from the NAPS2 study with both genotypic and phenotypic characteristics. For the nested analysis we compared patients with constant pain (n=504), constant-severe pain (n=450) and severe pain (n=727) with the patients who did not meet the categorical criteria. The goal of our analysis pipeline is to identify genetic variants that potentially alter expression of genes that have a biologically plausible mechanism of causing a more severe pain experience. The lead SNPs associated with plausible pain genes for Constant Pain, Constant-Severe Pain and Severe Pain from the initial GWAS/FUMA analysis are highlighted here as candidates for future evaluation. The complete analysis results are in Supplemental Information (as highlighted below).

### GWAS/FUMA

#### Constant pain

Manhattan plots and lead SNPs are shown for the GWAS results for constant pain (Table 2; Supplemental Fig. S1, S2 and Table S4), constant-severe pain (Table 3; Supplemental Fig. S3, S4 and Table S5) and severe pain (Table 4; Supplemental Fig. S5, S6 and Table S6). As expected, none of the 7745,456 SNPs tested reached independent genome-wide significance (p<5×10^−8^), but there were many suggestive significant loci with p<1×10^−5^. The lower threshold was chosen as a screening tool for *cis*-acting elements (e.g. genes within the same locus) noting that annotating the closest gene to a SNP is correct about 70% of the time^51–53^ and that *post-hoc* candidate gene selection would be applied using a literature search. The QQ plots also had low tails, as expected, since dominant genetic effects from monogenetic disorders were not expected (i.e. genetic variants are only manifest in combination with several other factors or only in specific conditions) and the complex association data likely contains false negatives (see QQ plots in Supplemental Figs. S2, S4, and S6).

GWAS/FUMA identified 13 genomic loci with 13 independent lead SNPs meeting suggestive significance in constant pain (Table 2, Supplemental Table S7). A review of the nearest gene(s) revealed multiple candidate genes associated with the constant pain phenotype (Synaptoporin [*SYNPR*], Neurotrophin 3 [*NTF3*], SLIT And NTRK Like Family Member 6 [*SLITRK6*]).

##### SYNPR.

The variant rs2060757C>T (MAF T=0.364 Allele Frequency Aggregator [ALFA] European^54^) is on chromosome 3 and intronic to *SYNPR,* which codes for synaptoporin, an intrinsic membrane protein of small presynaptic vesicles in neuron projections.^55–58^ Central expression of synaptoporin consistently represents synaptic terminations of peripheral afferents that include nociceptive Aδ- and C-fibers projecting to the dorsal horn.^55,56^ Thus, genetically altered expression or function of synaptoporin represents a plausible mechanism for future studies of constant pain patterns in humans.

*NTF3* and the BDNF signaling pathway. A chromosome 12 locus defined by rs10492094G>T (MAF T=0.324 ALFA European) is upstream to *NTF3,* which codes for neurotrophin 3 (NT3). NT3 is a neuronal growth factor that regulates the development, function and repair of the nervous system.^59^ NT3 is upregulated in the presence of inflammatory cytokines such as IL-1β or TNF-α, and stimulates nerve growth in cell cultures.^60^ NT3 binds to the receptor tyrosine kinase TrkC; whereas, nerve growth factor (NGF) binds to TrkA and brain-derived neurotrophic factor (BDNF) and neurotrophin 4 (NT4) bind to the TrkB receptor.^61^ While Trk receptors are primarily expressed in neurons in the CNS and dorsal root ganglia (DRG)^62^, another lower affinity neurotrophin receptor, p75 (p75NTR), is more widely distributed, with expression on pancreatic, neural, immune and Schwann cells. Of note, a subset of chronic pancreatitis patients who undergo surgical resection of the pancreas have marked neural hypertrophy that is associated with severe pain, while other CP patients do not exhibit these changes for unknown reasons.^63,64^

The regulation of neural signaling by NT3 is complex, as NT3 binds to TrkA and TrkB, and with higher affinity to TrkB than BDNF.^62^ In rodents, elevated NGF and BDNF are associated with neuropathic pain; whereas NT3 generally appears to alleviate neuropathic pain.^61^ DOK6 (Docking Protein 6) (below) is important in transport of TrkC along nerves^65^, and may therefore contribute to neuropathic pain. In an experimental model of diabetic neuropathy that causes reduction in NT3, administering NT3 moderately improved axonal disruption.^66^ Furthermore, we previously identified variants associated with *BDNF* linked to constant pancreatic pain and general anxiety disorder using a candidate gene approach.^14^ The previously identified SNP rs1491851T>C has an eQTL for BDNF Antisense RNA (*BDNF-AS*), a long noncoding antisense RNA transcript with highest expression in the spinal cord, followed by brain and peripheral nerves.^14,67^ This antisense RNA may be a negative regulator of BDNF expression.^68^ Thus, genetic variants near *NTF3* are plausible candidates for differences in patient pain experience linked to variant neuronal response to recurrent and chronic pancreatitis, possibly due to dysfunction of TrkB/BDNF and NT3/TrkC transport (DOK6) in neuropathic pain conditions.

#### Serum BDNF levels in patients with painful chronic pancreatitis

**Fig. 4.**
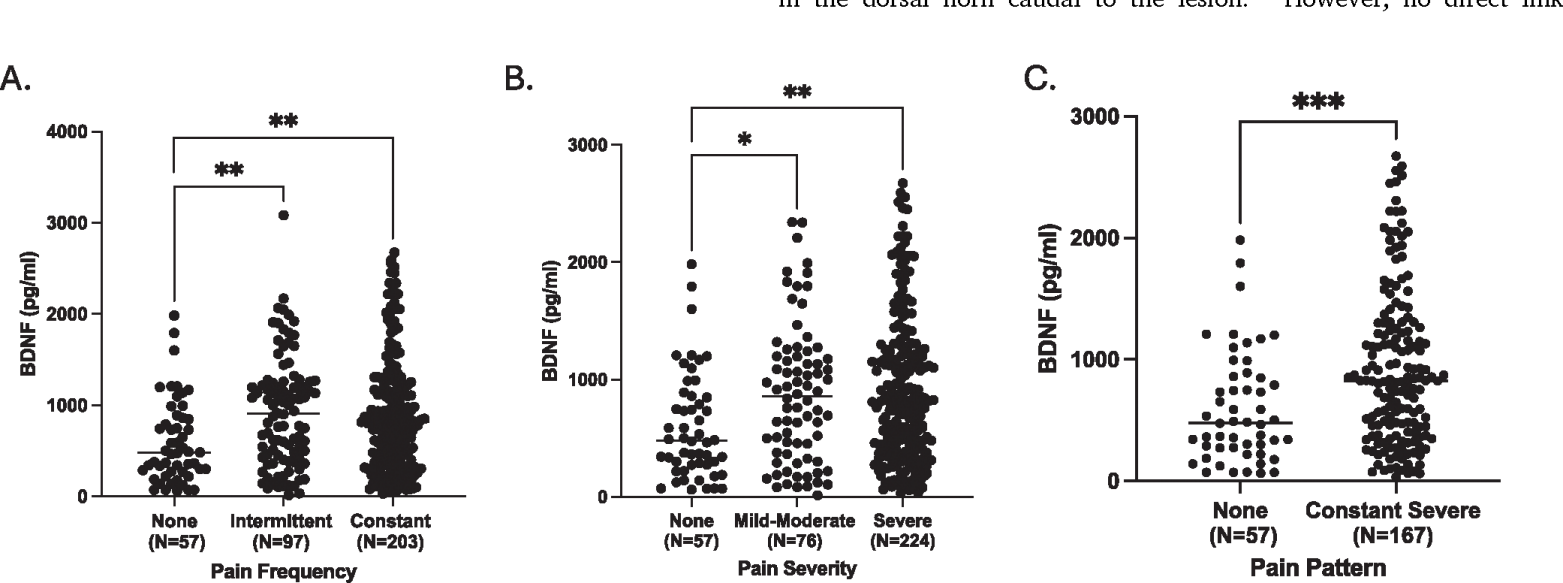
Serum BDNF levels in individuals with CP. Subjects with pain regardless of severity (A) or frequency (B) have significantly higher levels of serum BDNF compared to those with painless CP. Data compared by kruskal-wallis test. C) Subjects with constant, severe pain have significantly higher levels of serum BDNF compared to those with painless CP. Data compared by Mann-Whitney test. *0.05, **0.01, ***0.001.

Based on the link between NT3 and the BDNF pathway, we sought to test whether serum BDNF levels were altered in pancreatitis pain patients. In an independent cohort of individuals from the PROCEED study^49,50^, we found that serum BDNF levels were indeed significantly upregulated in subjects with painful chronic pancreatitis as compared to those with nonpainful CP (Figure 4). These data are consistent with predictions of the effects and direction of genetic changes in our patients with painful chronic pancreatitis.

##### SLITRK6.

An intergenic SNP on chromosome 13, rs117027346C>T (MAF T=0.036 ALFA European) is near *SLITRK6*, which codes for SLIT and NTRK-like protein 6 precursor. Rs117027346 is a member of a very large haplotype that spans the *SLITRK6* gene. The protein shares homology with Trk neurotrophin receptors (noted above) and has been associated with hearing and vision.^69,70^ There are no eQTLs listed on GTEx, but *SLITRK6* may be part of a co-expression network involved in voluntary movement and associated with neuropsychiatric phenotypes in mice.^71^ In neuronal cell cultures derived from human iPS cells, *SLITRK6* expression responded to zonisamide, an antiseizure drug being evaluated for neuropathic pain.^72^ Furthermore, survival of dopaminergic neurons was associated with *SLITRK6* expression levels.^73^

#### Constant-severe pain

In constant-severe pain, GWAS/FUMA identified 13 genomic loci and 14 independent lead SNPs associated with the phenotype (Table 3, Supplemental Table S8). Our analysis identified *SYNPO* (Synaptopodin) as a plausible candidate gene associated with the constant-severe pain phenotype and *RGMA* (Repulsive Guidance Molecule BMP Co-Receptor A) as a candidate for neuropathic pain. Other candidate genes that are not discussed included Tensin 3 (*TNS3*), a chromosome 7 gene with lead SNP rs334527 in the intergenic region and a haplotype affecting DNAse exposure in pancreatic cell (DNAse identifies cell subtype-specific regions of chromosomal DNA with limited histone protection that are exposed to regulatory elements, nucleosome occupancy and transcription factor binding).

##### SYNPO.

A chromosome 5 SNP, rs11745888C>T (MAF T=0.439 ALFA European), is annotated to the *SYNPO* gene that codes for synaptopodin. The splicing QTL (sQTL) for rs11745888C>T in GTEx is for *SYNPO* with p=7.4×10^−6^ in tibial nerve (https://www.gtexportal.org/home/snp/rs11745888).

Synaptopodin is expressed in kidney pseudopodia and the nervous system where it is essential for spine formation in telencephalic neurons.^74^ It also plays a role in epithelial cell apical stress biology.^75^ Elramah et al.^76^ recently demonstrated in a mouse model of cancer pain the upregulation of synaptopodin by downregulation of miR-124, an endogenous inhibitor of synaptopodin. Increase synaptopodin correlated with severe pain that was alleviated by intrathecal miR-124 infusion. While miR-124 may have additional targets^77^, the current association study suggests altered expression of *SYNPO* as a good candidate mechanism for differential pain experiences in pancreatitis patients.

##### RGMA.

A chromosome 15 SNP, rs7167068A>T (MAF T=0.475 ALFA European), is intronic to LOC105370982 (uncharacterized) and 260 kb 3′ of the Repulsive Guidance Molecule BMP Co-Receptor A RGMa gene (*RGMA*). RGMa binds to the Neogenin receptor resulting in axon growth inhibition and immune regulation.^78–80^ During embryonic development RGMa regulates axonal guidance, differentiation of neural stem cells into neurons, and the survival of these cells.^81^ *RGMA* is also upregulated after neuronal injury.^81^ Rats with traumatic spinal cord injury exhibit reduced neuronal survival, plasticity of descending serotonergic pathways and corticospinal tract axonal regeneration; these features were restored by treatment with anti-RMGa antibodies.^79^ Anti-RGMa also attenuated neuropathic pain behavioral responses and reduced activated microglia and calcitonin gene-related peptide (CGRP) expression in the dorsal horn caudal to the lesion.^79^ However, no direct link between rs&167068A>T and *RGMA* expression was identified, and further research is needed on this candidate gene.

#### Severe pain

Severe pain had 11 genomic loci identified by GWAS/FUMA with 12 independent lead SNPs meeting suggestive significance (Table 4, Supplemental Table S9). Candidate genes for severe pain based on GWAS results included the *REG* (Regenerating Family Member) gene cluster, *COBL* (Cordon-Bleu WH2 Repeat Protein), *LOC101927588 / TMEM65* (Transmembrane Protein 65), *RBFOX1* (RNA Binding Fox-1 Homolog 1), *DOK6* (Docking Protein 6), *LDLR* (Low Density Lipoprotein Receptor).

*REG* gene cluster. The chromosome 2 SNP rs1915703G>A (MAF A=0.303 ALFA European) is in an intergenic region close to, and with known eQTLs with two adjacent genes, ENSG00000234877.2 (*AC092660.1* [Clone-based (Vega) gene]) and ENSG00000214429.3 (*CYCSP6* [CYCS Pseudogene 6], one of many cytochrome C pseudogenes). Both are expressed in testes and to a small degree in the brain. The functions are unknown.

The rs1915703G>A variant is part of a common haplotype that is loosely linked with the *REG* gene cluster (Regenerating Family Member [REG] 1 Alpha [*REG1A*], REG 1 Beta [*REG1B*], REG 3 Alpha [*REG3A*], REG 3 Gamma [*REG3G*]) 421 K downstream. Linkage was found with multiple haplotypes having eQTLs for *REG1B* tagged by the rs61448477 haplotype (R^2^ 0.0041. D′ 0.0908 p<0.05) (plus *REG1P* and *REG3A* genes), and the rs1448213 SNP (R2 0.004, D′0.099, p-value <0.05), and *REG3G* tagged by rs283832 haplotype (R^2^ 0.0038, D′ 0.2087 p<0.05) and the rs1522857 SNP (R^2^ 0.0049, D′ 0.19 p-value <0.05).

The Regenerating Family Member (*REG*) genes are highly expressed in the pancreas, with *REG1A* also moderately expressed in the distal small intestine. REG gene products have been called pancreatitis-associated proteins, pancreatic stone proteins, lithostathine and others with multiple names for the same gene product and inconsistent number between genes in mouse and human. The REG proteins are multifunctional proteins that were initially believed to prevent pancreatic intraductal stone formation and later found to have antimicrobial activity, to be important for beta cell survival, regeneration, T cell regulation and M1/M2 macrophage polarization, stellate cell activation and proliferation, anti-cancer activities and other actions.^82–87^

*REG* gene products are known neurotrophic factors for motoneurons^88^, and are upregulated in the CNS following injury or disease where they have strong neuroprotective/neuroregenerative effects.^89^ In the pancreas, *REG3A* expression by stressed acinar cells is central to perineural invasion of pancreatic cancer^90^ and as such, may contribute to severe cancer pain. After spinal cord injury in rats, RGMa blocking antibodies promoted neuronal survival, and enhanced the plasticity of descending serotonergic pathways and corticospinal tract axonal regeneration.^79^ In mice with peripheral nerve injury, Reg3b (*REG3A* in humans) is transported to the spinal cord where it activates spinal microglia.^91^ Reg3b appears to maintain neuropathic pain by proinflammatory effects on microglia.^91^ Further studies on the effect of variants in specific REG genes related to pancreatic inflammation (with or without cancer) and neuropathic pain are needed.

##### COBL.

The chromosome 7 SNP rs757323G>A (MAF G=0.484 ALFA European) is 6 kb 3′ of or intronic to *COBL* (reverse direction). The cordon-bleu WH2 repeat protein regulates the assembly of intestinal microvilli^92^, neuron morphogenesis and promotes branching of axons and dendrites.^93–95^ *COBL* is highly expressed in the brain, muscles and peripheral nerves with low expression in the pancreas. An eQTL for rs757323 links to *COBL*.^96^ One GWAS study of subjects of European ancestry identified several SNPs near *COBL* associated with PTSD^97^, but it has not previously been associated with pain making it an interesting gene to consider in the future.

##### TMEM65 - RP11–37N22.1 loci.

Chromosome 8 loci are tagged by rs12548675T>C (MAF T=0.233 ALFA European) that is intronic to uncharacterized *RP11*–*37N22.1*(*LOC101927588*). This SNP has no eQTLs on GTEx or HaploReg and is not part of a haplotype block with regulatory SNPs.^96,98^ However, the closest protein-coding gene, *TMEM65,* is about 95 kb downstream of rs12548675. *TMEM65* codes for transmembrane protein 65, a critical mitochondrial membrane gene linked to the sodium-calcium exchanger that protects cells from necrotic death due to calcium overload.^99^ It is highly expressed in brain and muscle. In one case study a patient with homozygous pathogenic *TMEM65* gene mutations suffered from severe mitochondrial encephalomyopathy (including microcephaly, mutism and global developmental delay) with seizures and developmental regression at age 3 years.^100^ In a GWAS study *TMEM65* variants were associated with “fear of pain”^101^ and were differentially methylated in chronic widespread pain syndrome^102^ making it an interesting candidate for future studies.

##### RBFOX1.

A locus on chromosome 16 includes two independent SNPs linked to *RBFOX1*. rs34109083A>G(MAF G=0.086 ALFA European) is a tag-SNP for a large haplotype spanning the entire *RBFOX1* gene.^58,98^ In addition, rs67176054G>A (MAF A=0.0017 ALFA European) is an intronic variant in *RBFOX1*. There are no eQTLs for SNPs in the tagged haplotype but there are extensive changes in DNA motifs at promoter and enhancer histone marks (HaploReg V4.1).^98^ Likewise, there are no eQTLs for rs67176054, but the variant changes a SMAD3 binding motif.^98^

*RBFOX1* codes for RNA Binding Fox-1 Homolog 1 (RBFOX1), an RNA binding protein that regulates alternative splicing events by binding to 5′-UGCAUGU-3′ elements. *RBFOX1* is highly expressed in brain (especially frontal cortex), muscle, heart and other tissues such as the kidney.^96^ RBFOX1 appears to modify the post-transcriptional landscape of gene splice variants in response to stress as demonstrated in human renal proximal tubular epithelial cells (HK-2 cells) where exogenous RBFOX1 inhibited inflammation and oxidative stress to reduce hypoxia/reoxygenation-induced apoptosis of HK-2 cells.^103^ In the brain, RBFOX1 modifies the activity of synaptic regulators in response to neuronal activity, keeping excitability within healthy domains.^104,105^ For example, it modifies expression of a TrkB isoform, reducing binding of BDNF^106^ (see discussion of BDNF biology under NTF3). RBFOX1 also modifies the transcriptional corepressor Lysine Specific Demethylase 1A (*LSD1/KDM1A*) isoforms. *LSD1* is a homeostatic immediate early gene (IEG) regulator that plays a relevant part in the environmental stress--response.^104^ Based on several genetic associations of the alternative splicing regulator *RBFOX1* with psychiatric conditions and biological connections with *LSD1* and IEGs, Forastieri et al^104^ concluded that homeostatic unbalance linked to these factors provides a neuronal signature of stress-associated psychiatric conditions. Indeed, genetic variants linked to *RBFOX1* have been associated with nicotine dependence^107,108^, addiction to cocaine in mice^109^, neuroticism, MDD^110^, autism^111,112^ and schizophrenia.^107^ To our knowledge, our study is the first to associate variants that are associated with *RBFOX1* with severe pain experience in pancreatitis.

*DOK6* is an important gene associated with axon guidance and function and discussed in TWAS and colocalization results (below). A lead SNP on chromosome 19, rs35878749G>A (MAF A=0.353 ALFA European) is intronic to *LDLR* and is an eQTL for *SPC24* (SPC24 Component Of NDC80 Kinetochore Complex), a gene with no clear link to pain. *LDLR* was identified as a candidate in TWAS and is discussed below.

### TWAS

There was one gene that reached Bonferroni corrected significance (p-value < 2.8×10^−6^) from the TWAS S-MultiXcan in constant (Supplemental Table S10) and constant-severe pain (Supplemental Table S11), MAML1 (Mastermind Like Transcriptional Coactivator 1, p-value 2.07e-7, and 4.99e-8 respectively). *CTRC* (Chymotrypsin C, p-value 2.45e-5) and *NEURL3* (Neuralized E3 Ubiquitin Protein Ligase 3, p-value 9.28e-6) met suggestive significance (p-value < 1×10^−4^) for constant pain (Figures 1 and 2). *CTRC* (p-value 4.5e-5), *HSF2* (Heat Shock Transcription Factor 2, p-value 5.85e-6) and *ZNF385D* (Zinc Finger Protein 385D, p-value 8.25e-5) met suggestive significance for constant- severe pain (Fig. 2, Supplemental Table S11). LDLR (p-value 6.53e-5) and DOK6 (p-value 7.5e-5) met suggestive significance for severe pain (Fig. 3, Supplemental Table S12).

Each of the TWAS identified genes is discussed below, including information aggregated from a post hoc literature search supporting the candidacy of each gene. Each reported gene shows differential expression across all tissues associated with the pain phenotype.

### MAML1

The results of TWAS predict *MAML1* to be differentially expressed in constant pain subjects (greatest GTEx eQTL effect in the heart), and in constant-severe pain subjects (greatest effects seen in the cerebellar hemisphere of the brain). *MAML1*, mastermind like transcriptional coactivator 1, codes for the human version of the *Drosophila* mastermind protein, which is involved with Notch signaling.^113^ *MAML1* is critical in protein translation and regulation in humans, affecting the NOTCH signaling pathway, Hippo signaling, NF-κB, and Sonic Hedgehog signaling.^57,114^ *MAML1, MAML2* (Mastermind Like Transcriptional Coactivator 2) and *MALM3* (Mastermind Like Transcriptional Coactivator 3) are functionally similar.^115^ Johnston et al^20^ identified a risk haplotype tagged by rs13136239 in the *MAML3* introns associated with multisite chronic pain in UK Biobank (p=3.6e^−8^). The mechanism(s) linking *MAML1* and *MAML3* to pain are unknown, but multiple plausible mechanisms have been proposed.^20^, e.g., a link between *MAML1* and NF-κB signaling may dysregulate immune balance in the pancreas.

### CTRC

Our TWAS suggests that *CTRC* is differentially expressed in patients with constant pain and constant-severe pain with the greatest effect seen in the pancreas. *CTRC* codes for chymotrypsin C, a pancreatic digestive enzyme that plays an important role in protecting the pancreas from trypsin-associated injury by cooperating in the proteolytic destruction of the trypsin molecule.^116^ *CTRC* is expressed almost exclusively in the pancreas. Loss of function or lowered expression of *CTRC* is a major risk factor for chronic pancreatitis, with the most commonly seen risk haplotype defined by rs497078C>T (p.G60G) (MAF T=0.092 ALFA European), which is strongly associated with reduced function (p = 3.2×10^−14^).^58,96,117^ Thus, it is plausible that constant and constant-severe pancreatic pain are associated with continued, subclinical, trypsin-associated inflammation (see also Colocalization results, below).

Of note, differential expression of *CTRC* is suggestively associated with both constant and constant-severe pain, whereas variants altering *SPINK1* (Serine Peptidase Inhibitor Kazal Type 1) expression (haplotype tagged by rs17107315T>C [MAF C=0.0098 ALFA European] p. Asn34Ser/N34S) coding for another trypsin inhibitor, are not. Our study is likely underpowered to detect effects of altered *SPINK1* expression because the MAF of the common risk haplotype of *SPINK1* is 10% of the common *CTRC* risk haplotype mentioned above.

### NEURL3

TWAS predicts differential expression of *NEURL3* in patients with constant pain with greatest effect in GTEx seen in the substantia nigra of the brain. *NEURL3,* neuralized E3 ubiquitin protein ligase 3 formally known as *LINCR*, is involved in protein ubiquitination and is primarily expressed in salivary glands and pancreas.^57,96,113^ NEURL3 is also involved in cellular mechanisms involved in spinal development.^118,119^ Increased expression of *NEURL3* is reported in lung tissue in response to inflammation from endotoxemia.^120^ Genetically altered expression of *NEURL3* in response to inflammation may be important in the pathophysiology of patients with constant pancreatitis pain.

### HSF2

TWAS suggests differential expression of *HSF2* in patients with constant-severe pain with the strongest effect in GTEx reported in “skin not sun exposed suprapubic”. *HSF2* encodes a heat shock factor (HSF) protein, heat shock transcription factor 2, and is highly expressed in the brain.^113^ HSF2 is a transcription factor involved in chromatin condensation, regulation of the cell cycle^121^ and is activated by hemin rather than heat.^121^ Additionally, HSF2 also activates the transcription of genes in response to oxidative stress, similar to what is seen in acinar and duct cells^122^ making it another candidate for studies of pancreatitis pain (see also Colocalization results, below).

### ZNF385D

TWAS also predicted differential expression of *ZNF385D* in patients with constant-severe pain with the most significant GTEx effect size seen in the aorta although it is primarily expressed in the brain.^57,113^ *ZNF385D* codes for zinc finger protein 385D. A GWAS of placebo and duloxetine response in MDD, suggested differential effectiveness of duloxetine based on the *ZNF385D* genotype (rs4261893; β=−0.46, p=1.55×10^−5^).^123^ These data suggest that *ZNF385D* genotypes may be linked to the stress-associated psychiatric disorder MDD and may predict drug effectiveness in some patients with severe pancreatitis pain.

### LDLR

Differential expression of *LDLR* was predicted in patients with severe pain by the current TWAS with greatest effect seen in arteries. *LDLR* codes the low density lipoprotein receptor which is normally a cell surface protein.^57,113^ Mutations in this gene are associated with familial hypercholesterolemia.^124^ The link between *LDLR* genotypes and neuropathy with severe pain is not clear, but abnormal lipid metabolism is associated with neurologic conditions such as Alzheimer’s disease and *RGMA* (Repulsive Guidance Molecule BMP Co-Receptor A) genetic variants (see above) also affects lipid levels.^125^ *LDLR* expression in the forebrain may affect BDNF levels^126^ possibly linking *LDLR* variants to psychiatric stress disorders. ^127,128^

### DOK6

Differential expression of *DOK6* was predicted in patients with severe pain in pancreatitis with greatest effect in nerve tissue. *DOK6*, docking protein 6, is involved in the RET receptor tyrosine kinase signaling cascade and is expressed in brain and peripheral neuron populations.^113^ RET signaling is key to axon guidance, neuron development and functional properties.^129^ DOK6 acts as an adaptor protein for selectivity-mediated neurotrophic signal transduction and retrograde transport for TrkC and Ret but not for TrkA and TrkB.^65^ The effect of variant DOK6 genotypes on experience of severe pancreatic pain is not known, but likely plays a major role in multiple central and peripheral neural subtypes (see also Colocalization results, below).

#### Colocalization

Colocalization was performed on the gene-tissue pairs identified from the TWAS (Table 5, Supplemental Tables S10, S11, S12). For constant pain, the non-significant *CTRC* GWAS signals colocalized with the eQTL in pancreas tissue (Supplemental Fig. S7). In constant pain, *CTRC* had a PP.H4 0.73 and a PP.H3 0.02, suggesting that the signals colocalize to one SNP. Additionally, the sum of PP.H0-PP.H2 (0.25) was less than 0.3, indicating that, even though the GWAS signals for *CTRC* were not significant at our cutoff level, these signals were likely due to the same SNP as the eQTL.

The eQTL and constant-severe pain GWAS signals colocalized in *HSF2* (PP.H4 0.7, PP.H3 0.06, PP.H0+PP.H1+PP.H2 0.24) in skin not sun exposed suprapubic (Supplemental Fig. S8). Finally, the signals from *DOK6* (PP.H4 0.98, PP.H3 0.01, PP.H0+PP.H1+PP.H2 0.01) associated with severe pain colocalize with nerve tibial tissue (Supplemental Fig. S9).

#### Integrated model of pain mechanisms

**Fig. 5.**
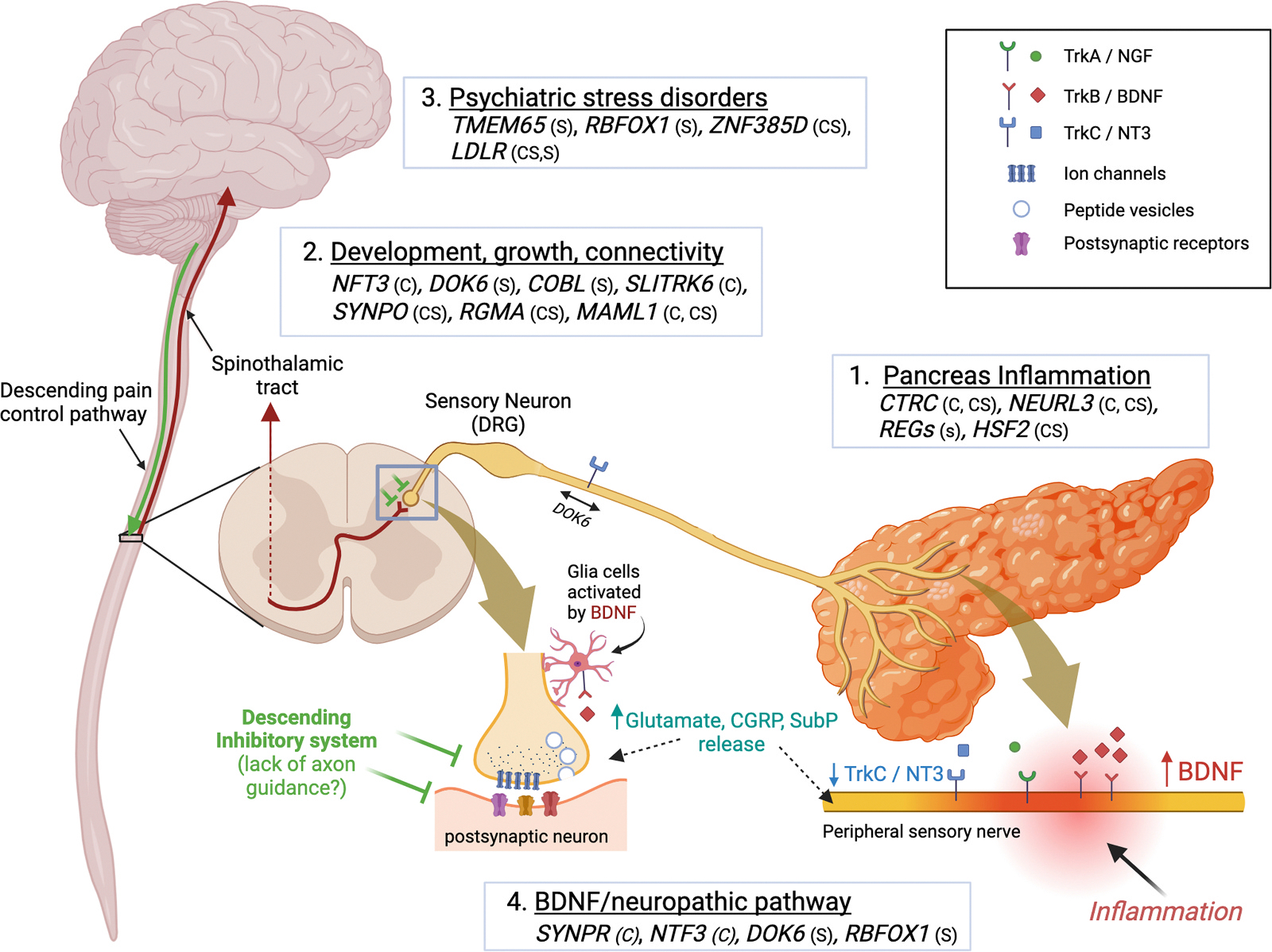
Pancreatic pain syndrome model. Pancreatitis pain is initiated with injury and inflammation in the pancreas and transmitted via sensory nerves with cell bodies in the dorsal root ganglia (DRG) to second order neurons in the spinal cord. Pain signals are transmitted up the spinothalamic tract to the thalamus where third-order neurons transmit signals to other parts of the somatosensory cortex and limbic system. A descending pathway originates in the periaqueductal gray matter, project to the medulla and descend to the spinal cord segment receiving pain signals allowing modulation of the pain response. The 4 major systems identified by candidate pain genes are numbered along with gene codes (see text) and type of pain (c, constant; cs, constant-severe; s, severe). Neuropathic pain is associated with changes in the BDNF system and associated with increased sensory nerve release of glutamate, CGRP, substance P (SubP) both in the periphery (linked to neuroinflammation) and centrally. Figure created with BioRender.com.

To comprehend the implications of the above findings, we organized the identified genes according to predicted mechanism and function into 4 categories, (1) pancreatic inflammation; (2) development, growth and connectivity; (3) psychological stress disorder genes; and (4) dysfunction of the BDNF pathway known to be associated with neuropathic pain (Fig. 5). In some cases, a gene may be associated with more than one system, such as *DOK6* and *NTF3.* We noted how select genes may impact the ascending pain pathway from primary afferents in the pancreas, to the spinal cord and brain, with additional factors that impact pain regulation (e.g. descending serotonergic pathways and *REG3*) and pain perception (e.g. psychiatric stress disorders).

## Discussion

Pancreatitis pain can be devastating both mentally and physically, and difficult to treat.^1,11–14^ The complexity of pancreatic disease, the variability of pain experience that is often independent of imaging findings and the failure of single, traditional approaches to provide predictable and lasting relief indicate that individualized treatments targeting the true pain mechanism(s) are needed. In this exploratory study we discovered that pancreatitis patients with pathologic pain syndromes with abnormal chronicity and/or severity share multiple genetic risk loci that overlap with known pain syndromes from other anatomical sites and with biological models of pain. This work adds new loci to the previously identified stress-related psychiatric disorder loci, moving a step closer to new precision treatments.^11,13,14^ Predicting altered drug responses to zonisamide^72^ and duloxetine^123^ based on genotyping loci from this study may prove, with confirmation studies, to be immediately applicable to better pain management in pancreatitis patients and other pain disorders.

Using a combined lower than genome-wide significant p-value screening (e.g. p<1×10^−5^) with a post hoc candidate gene selection method we replicated known pain-associated loci and identified multiple plausible genes within pain-associated loci where dysfunctional expression mechanisms (failure to be expressed in the right specialized cell, in the right amount, at the right time) or protein dysfunction of the candidate genes could predispose to one or more type(s) of pain syndromes. We found 3 candidate genes within 13 pain-associated loci for constant pain (23.1%), 3 genes within 13 loci for constant-severe pain (23.1%), and 4 genes within 11 loci for severe pain (36.4%). Further analysis using TWAS to verify genes in *cis* and identify additional genes in *trans* to the tag-SNPs identified 7 candidate pain-associated genes, strengthening the plausibility of candidates using this approach. Furthermore, we observed that the three pancreas genes associated with chronic inflammation (*CTRC, NEURL3, HSF2*) were within the constant (chronic) pain phenotype, while the single injury-response gene (*REG*) was identified in the pain severity phenotype, as expected within pancreatic biologic mechanism. In contrast, all four psychiatric stress-related genes were associated with a more severe pain experience. This represents the first systematic genetic analysis of pancreatitis pain loci, complementing and extending the candidate gene studies for depression, anxiety and PTSD.^13,14^ More compelling are the 4 genes linked to the BDNF neuropathic pain pathway (*SNYPR, NTF3, DOK6* and *RBFOX1*) with elevated BDNF levels in pancreatic pain patients. Thus, even within the general pancreatitis phenotype of pain syndromes, the genotypes correlated with the expected subtype of pain and with a serum pain biomarker.

Although the exact biological mechanisms of chronic pain are unknown, some known pathways include: GABAergic, catecholaminergic, cytokines, growth factors, serotonergic, estrogenic, glutamatergic, proteinases, neurogenesis, nervous-system development, and neural connectivity.^19,20,130^ The new candidate genes fall into four well defined groups: (1) pancreatic inflammation; (2) development, growth and connectivity (including injury repair and stress genes); (3) psychological stress disorder genes (linked to MDD, GAD and PTSD); and (4) dysfunction of the BDNF pain pathway known to be associated with neuropathic pain (Fig. 5). Many of the candidate genes discussed above are involved in nervous-system development, growth, and connectivity (*NFT3*, *DOK6*, *COBL*, *SLITRK6*, *SYNPO, RGMA and MAML1*). This is by far the most complex category as some genes likely play a role in development as well as regenerative and phenotypic responses to injury and inflammation. With respect to pain syndromes in pancreatic inflammation, it is known that many patients have dysfunctional descending pain control mechanisms^24^, and failure of specific regulatory nerves to connect with ascending pain pathways in the spinal cord would result in failure to adapt to peripheral pain signals.

The *BDNF* pathway appears to be especially important for pancreatitis pain as well as anxiety.^14^ In rodent models of chronic pancreatitis, BDNF is upregulated and appears to mediate pain-associated behavior.^131^ In humans, anatomical studies of neurotrophin/growth factor expression demonstrated that BDNF is upregulated in pancreatic tissue of patients undergoing surgery for severe pain from chronic pancreatitis.^132^ Our study provides new insights into the variability of painful human chronic pancreatitis and may serve to identify subsets of patients where altered BDNF biology is contributing to a more severe pain experience than exacted by pancreatic inflammation alone. The findings in this study confirm and extend the concept of heterogeneity of etiologies contributing to pain in chronic pancreatitis^24,133–135^ and support the precision medicine view that optimal treatment will require specific treatments targeting the dysfunctional mechanism.^11,136,137^ The genes and pathways identified here overlap with the findings of pain genetic studies in other diseases and injury syndromes, indicating common central and peripheral nervous system and inflammatory system problems with a genetic basis, and that may respond to similar targeted treatments. Thus, future research is needed to examine whether underlying genetic risks predict biochemical and physiological biomarker signals and effective pain control treatments.

This study had several notable strengths. First, the NAPS2 data set includes detailed information on the type, severity and trajectory of pancreatitis patients, as well as deep phenotyping on pain onset, character, severity and chronicity. ^1,9,12–14,138–140^ Second, the approach taken here is a highly innovative screening project to test the hypothesis that differences in pancreatitis patient’s highly variable pain response to pancreatic inflammation has central, as well as peripheral links to genetic variants. Taking GWAS results and incorporating functional biological information using TWAS increases the power of the results and ability to identify genetic findings that would be underpowered in GWAS alone. The addition of statistical colocalization tests confirms that the overlap of the GWAS signal and the eQTL in a locus is not random and that they are not independent. To our knowledge, this is the first application of TWAS and colocalization to pancreatitis pain. Linking the finding of variants associated with the BDNF system with elevated BDNF levels in an independent cohort of subjects with definite CP, provides additional evidence for the application of these findings.

### Limitations

The findings were largely limited to existing data sets from the NAPS2 study that primarily consist of individuals of European Ancestry who were genotyped using a GWAS array that was also enriched in European variants.^3,141,142^ Additionally, this study is underpowered to discover additional important, but less common pain gene variants and reduce false discovery due to small sample sizes. In prior studies, candidate gene methods were used to alleviate low power issues. Here the extensive post-GWAS methods, TWAS and colocalization, were used to provide additional biologically informed results using the data available to us.

The TWAS uses expression data from GTEx^96^ to predict which genes may be differentially expressed in patients with more severe pancreatitis pain. GTEx uses tissues harvested postmortem to study gene expression.^96^ The “normal” expression that the prediction models use is therefore limited to the biological conditions of the tissues when they were harvested, which may not be an accurate representation of the expression profile of our patients. This is one reason that candidate genes from the GWAS are not identified by the TWAS. However, given the incorporation of biological information TWAS is better suited to predict candidate genes than an underpowered GWAS was able to detect.

Many of the loci that were statistically associated with RAP and CP pain did not have any obvious candidate genes present. Most loci contained non-coding RNAs with currently unknown function that many contribute to critical gene regulatory mechanisms that remain to be discovered. Other candidate genes within a pain locus did not have obvious pathogenic variants or eQTLs or were thousands of kb away from the lead SNP. In these cases, the candidate gene would be a false discovery. Nevertheless, the consolidation of multiple genes within four known pain pathways suggests that many of the findings are true positives.

A final limitation of this study is the lack of a replication cohort from a similar population and with similar rigorous criteria for patient ascertainment and phenotyping of pancreatitis and pain syndromes. Replication of candidate genes and variants (or haplotypes) in another study provides additional statistical evidence. Beyond this, future mechanistic studies are needed to better understand both the damaging biological mechanisms and potential treatments. Thus, despite numerous limitations the initial phase of recognizing the link between pathologic pancreatic pain experience and genetic variants in neuronal genes in the brain, spinal cord and peripheral nervous system is a major step forward.

### Conclusion

We used a novel GWAS/TWAS candidate gene approach to explore the presence of genetic variants within known pain systems that are associated with pain syndromes in patients with RAP and CP. Future studies are needed to validate and add additional risk variants and risk loci, and to begin developing better diagnostic tools and treatment strategies to improve the health and welfare of patients with RAP and CP suffering from distressing pain syndromes.

## Supplementary Material

1

Appendix A. Supporting information

Supplementary data associated with this article can be found in the online version at doi:10.1016/j.jpain.2024.104754.

## Figures and Tables

**Table 1:** Sample Sizes of Pain GWAS.

Pain	Variable	Cases	Controls	Total

**Constant**	**Sample Size**	504	750	1254
	**Etiology: Alcohol Alone**	165	149	314
	**Etiology: Alcohol Plus**	47	92	139
	**Etiology: Genetic**	56	64	120
	**Etiology: Idiopathic**	117	232	349
	**Etiology: Obstructive**	46	81	127
	**Etiology: Autoimmune**	10	16	26
	**Etiology: Hyperlipidemia**	23	21	44
	**Etiology: Gallstone**	10	31	41
	**Etiology: Medications**	3	5	8
	**Etiology: Other**	26	56	82
	**Etiology: Missing**	1	3	4
	**Sex: Male**	238	398	636
	**Sex: Female**	266	352	618
**Constant-Severe**	**Sample Size**	450	804	1254
	**Etiology: Alcohol Alone**	149	165	314
	**Etiology: Alcohol Plus**	42	97	139
	**Etiology: Genetic**	51	69	120
	**Etiology: Idiopathic**	100	249	349
	**Etiology: Obstructive**	44	83	127
	**Etiology: Autoimmune**	10	16	26
	**Etiology: Hyperlipidemia**	20	24	44
	**Etiology: Gallstone**	9	32	41
	**Etiology: Medications**	2	6	8
	**Etiology: Other**	22	60	82
	**Etiology: Missing**	1	3	4
	**Sex: Male**	210	426	636
	**Sex: Female**	240	378	618
**Severe**	**Sample Size**	727	527	1254
	**Etiology: Alcohol Alone**	219	95	314
	**Etiology: Alcohol Plus**	73	66	139
	**Etiology: Genetic**	81	39	120
	**Etiology: Idiopathic**	173	176	349
	**Etiology: Obstructive**	67	60	127
	**Etiology: Autoimmune**	12	14	26
	**Etiology: Hyperlipidemia**	29	15	44
	**Etiology: Gallstone**	19	22	41
	**Etiology: Medications**	3	5	8
	**Etiology: Other**	50	32	82
	**Etiology: Missing**	1	3	4
	**Sex: Male**	374	262	636
	**Sex: Female**	353	265	618

The sample sizes for cases and controls. Etiologies are included for reference purposes. Missing etiology was rare (<1%) and these patients were included in the case-control analyses.

**Table 2:** GWAS/FUMA Independent Lead SNPS for Constant Pain.

GenomicLocusRegion^a^	rsID	chr	pos	p^b^	nGWASSNPs^c^	nearestGene

1:40814528–40823404	rs4660406	1	40,823,404	3.69e–06	5	*SMAP2*
3:63441514–63455599	rs2060757	3	63,455,599	9.01e–06	5	***SYNPR**:SYNPR-AS1*
4:184484425–184505515	rs10009455	4	184,494,883	8.80e–06	16	*ING2*
5:49435222–49435222	rs149312484	5	49,435,222	3.05e–06	1	*EMB*
7:47565793–47575970	rs334527	7	47,567,227	1.51e–06	13	*TNS3*
8:138803133–138806916	rs66890414	8	138,803,658	8.64e–06	3	*FAM135B*
12:5441541–5487932	rs10492094	12	5478,148	3.52e–06	3	***NTF3***
13:20847066–20866839	rs9552131	13	20,855,444	3.17e–06	35	*GJB6*
13:86362179–86565426	rs117027346	13	86,362,179	7.69e–06	100	***SLITRK6***
13:103580361–103606829	rs701545	13	103,580,541	1.51e–07	3	*METTL21EP / POGLUT2*
16:81238750–81264177	rs111271001	16	81,259,428	6.19e–06	21	*PKD1L2 (GAN)*
19:295231–295295	rs734885	19	295,231	7.50e–06	2	*PPAP2C (PLPP2) / MIER2 (eQTL)*
20:62200860–62263747	rs6062978	20	62,256,590	8.20e–06	6	*GMEB2 (& eQTL)*

a chr:start-end based on hg19

b GWAS p value

c Number of GWAS SNPs in high LD with lead SNP

**Table 3:** GWAS/FUMA Independent Lead SNPs for Constant-Severe Pain.

GenomicLocusRegion^a^	rsID	chr	pos	p^b^	nGWASSNPs^c^	nearestGene

1:54896755–54922021	rs4927113	1	54,902,861	5.08e–06	15	*SSBP3*
3:148698474–148876261	rs58186391	3	148,839,366	1.54e–06	53	*HPS3*
5:49435222–49435222	rs149312484	5	49,435,222	9.35e–06	1	*EMB*
5:149954864–149990727	rs11745888	5	149,968,929	3.91e–06	40	***SYNPO***
6:122429305–122921183	rs9388097	6	122,885,461	9.60e–06	97	*PKIB*
6:122429305–122921183	rs76046919	6	122,903,206	2.73e–06	6	*PKIB*
7:47565793–47575970	rs334527	7	47,567,227	2.59e–07	13	*TNS3*
8:138803133–138806916	rs66890414	8	138,803,658	6.27e–06	3	*FAM135B*
11:116519655–116519655	rs516226	11	116,519,655	6.39e–06	1	*AP000770.1*
12:5284122–5315245	rs645410	12	5301,847	4.71e–06	17	*RP11–319E16.1*
12:12963744–12990341	rs17394079	12	12,990,341	8.41e–06	10	*DDX47*
14:46976743–46986881	rs7161256	14	46,976,743	1.68e–06	2	*LINC00871*
15:93892942–93908051	rs7167068	15	93,893,035	7.14e–07	4	***LOC105370982 / RGMA***
19:11221180–11232696	rs35878749	19	11,229,765	7.26e–06	10	***LDLR / SPC24 (eQTL)***

a chr:start-end based on hg19

b GWAS p value

c Number of GWAS SNPs in high LD with lead SNP

**Table 4:** FUMA Independent Lead SNPs for Severe Pain.

GenomicLocusRegion^a^	rsID	chr	pos	p^b^	nGWASSNPs^c^	nearestGene

1:213685950–213755621	rs530848	1	213,732,214	4.45e–06	27	*RPL31P13*
2:78764895–78837866	rs1915703	2	78,832,777	4.70e–06	11	*CYCSP6 (eQTL) / REG cluster*
3:109525798–109681921	rs75623530	3	109,672,395	4.33e–06	30	*MIR4445*
6:155022713–155160128	rs7771767	6	155,038,479	8.18e–06	76	*SCAF8*
7:51035899–51079151	rs757323	7	51,077,759	4.54e–06	6	***COBL***
8:125224719–125224719	rs12548675	8	125,224,719	1.39e–06	1	*TMEM65 / FER1L6*
9:139614170–139642961	rs2275160	9	139,621,168	6.76e–06	10	*SNHG7* (intronic)
16:7353976–7417955	rs67176054	16	7371,066	6.34e–07	17	***RBFOX1***
16:7353976–7417955	rs34109083	16	7380,549	1.12e–06	53	***RBFOX1***
18:67306031–67327598	rs11663004	18	67,324,345	8.75e–06	26	***DOK6***
19:11221180–11232696	rs35878749	19	11,229,765	9.45e–07	10	***LDLR***
19:27947716–28309577	rs62111935	19	27,992,394	7.79e–06	93	*LINC00662*

a chr:start-end based on hg19

b GWAS p value

c Number of GWAS SNPs in high LD with lead SNP

**Table 5:** Coloc Results for Pain GWAS.

Pain	Gene	Tissue	nsnps	PP.H0	PP.H1	PP.H2	PP.H3	PP.H4	Colocalization^a^

Constant	*MAML1*	Heart Left Ventricle	16	0.73	1.59e–03	0.24	4.87e–04	0.03	No
	*NEURL3*	Brain Substantia Nigra	1	1.00	3.38e–04	2.40e–03	0	8.13e–04	No
	***CTRC***	Pancreas	30	5.45e–03	3.60e-04	0.25	0.02	0.73	Yes
Constant-Severe	*MAML1*	Brain Cerebellar Hemisphere	1	1.00	1.24e–04	2.50e–04	0	3.10e–05	No
	***HSF2***	Skin Not Sun Exposed Suprapubic	69	0.05	0.01	0.18	0.06	0.70	Yes
	*ZNF385D*	Artery Aorta	51	6.52e–03	1.44e–04	0.69	0.02	0.29	No
	*CTRC*	Pancreas	30	0.01	2.70e–04	0.46	0.01	0.51	No
Severe	*LDLR*	Artery Tibial	1	1.00	1.16e–04	6.02e–04	0	6.97e–05	No
	***DOK6***	Nerve Tibial	17	2.07e–03	2.77e–03	9.00e–03	0.01	0.98	Yes

a Evidence of Colocalization taken to be PP.H4 > 0.5 and PP.H3 <0.5, PP.H0+PP.H1+PP.H2<0.3.

## Data Availability

Data is not publicly available as samples were collected before public availability was required and informed consent of some sites did not include necessary statements.

## for the NAPS2* study group.

### *NAPS2 Consortium

Stephen T. Amann, MD^5^; Peter Banks, MD^6^; Randall Brand, MD^1^; Darwin L. Conwell, MD MSc^6c^; Greg Cote MD MS^7d^, Christopher E. Forsmark, MD^8^; Timothy B. Gardner, MD^9^; Nalini M. Guda, MD^10^; Michele D. Lewis, MD^11^; Jorge D. Machicado, MD^1d^; Thiruvengadam Muniraj, MD, PhD^1f^; Georgios I. Papachristou, MD, PhD^1g^; Joseph Romagnuolo, MD^7h^; Bimaljit S. Sandhu, MD^12i^; Vikesh K Singh MD, MSc^13^; Stuart Sherman, MD^14^*; Adam Slivka, MD, PhD^1^, C. Mel Wilcox, MD^15j^;

*Deceased.

#### Affiliations

1. Department of Medicine,University of Pittsburgh, Pittsburgh, PA, USA.

5. North Mississippi Medical Center, Tupelo, MS, USA

6. Department of Medicine, Harvard University, Brigham and Woman’s Hospital, Boston, MA, USA

7. Division of Gastroenterology and Hepatology, Department of Medicine, Medical University of South Carolina, Charleston, SC.

8. Department of Medicine, University of Florida College of Medicine, Gainesville, FL, US

9. Section of Gastroenterology and Hepatology, Dartmouth-Hitchcock Medical Center, Lebanon, NH

10. Aurora St. Luke’s Medical Center, GI Associates, Milwaukee, WI, USA

11. Department of Medicine, Division of Gastroenterology and Hepatology, Mayo Clinic, Jacksonville FL, USA

12. Department of Medicine, Medical College of Virginia, Richmond, VA

13. Pancreatitis Center, Division of Gastroenterology, Johns Hopkins Medical Institutions, Baltimore, MD, USA.

14. Department of Medicine, Indiana University, Indianapolis, IN

15. Department of Medicine, University of Alabama at Birmingham, Birmingham, AL, USA

Current Address

c. Department of Internal Medicine, University of Kentucky College of Medicine, Lexington, KY

d. Division of Gastroenterology & Hepatology, Oregon Health & Science University, Portland, OR .

f. Department of Medicine, Yale University, New Haven, CT

g. Department of Internal Medicine, Division of Gastroenterology, Hepatology, and Nutrition, The Ohio State University Wexner Medical Center, Columbus, OH.

h. Ralph H. Johnson VAMC, Charleston, SC.

i. St Mary’s Hospital, Richmond, VA.

j. Orlando Health Digestive Health Institute, Orlando, FL.

## Glossary of Gene Names (requested by reviewers)

AC092660.1: Clone-based (Vega) gene
BDNF: Brain Derived Neurotrophic Factor
BDNF-AS: BDNF Antisense RNA
COBL: Cordon-Bleu WH2 Repeat Protein
CTRC: Chymotrypsin C
CYCSP6: CYCS Pseudogene 6
DOK6: Docking Protein 6
HSF2: Heat Shock Transcription Factor 2
KDM1A (formerly LSD1): Lysine Specific Demethylase 1A
LDLR: Low Density Lipoprotein Receptor
MAML1: Mastermind Like Transcriptional Coactivator 1
MAML2: Mastermind Like Transcriptional Coactivator 2
MAML3: Mastermind Like Transcriptional Coactivator 3
NEURL3: Neuralized E3 Ubiquitin Protein Ligase 3
NTF3: Neurotrophin 3
RBFOX1: RNA Binding Fox-1 Homolog 1
REG: Regenerating Family Member gene cluster
REG1A: Regenerating Family Member 1 Alpha
REG1B: Regenerating Family Member 1 Beta
REG3A: Regenerating Family Member 3 Alpha
REG3G: Regenerating Family Member 3 Gamma
RGMA: Repulsive Guidance Molecule BMP Co-Receptor A
SLITRK6: SLIT And NTRK Like Family Member 6
SPC24: SPC24 Component Of NDC80 Kinetochore Complex
SPINK1: Serine Peptidase Inhibitor Kazal Type 1
SYNPO: Synaptopodin
SYNPR: Synaptoporin
TMEM65: Transmembrane Protein 65
TNS3: Tensin 3
ZNF385D: Zinc Finger Protein 385D
