# Supplementary material for: Genetics of constant and severe pain in the NAPS2 cohort of recurrent acute and chronic pancreatitis patients
Source: J Pain. Author manuscript; Available in PMC 2025 Jun 26. (PMC12199748; doi:10.1016/j.jpain.2024.104754)
Supplement: 1 [file NIHMS2087673-supplement-1.docx]

## Supplemental Tables and Figures

**Title**: Genetics of Constant and Severe Pain in the NAPS2 Cohort of Recurrent Acute and Chronic Pancreatitis Patients.

**Authors:** Ellyn K. Dunbar, MS, PhD; Phil J. Greer, MS; Jami L. Saloman, PhD; Kathryn M. Albers, PhD; Dhiraj Yadav, MD, MPH and David C. Whitcomb, MD, PhD for the NAPS2* study group.

Table S1

Demographics of Constant Pain

| **Variable** | **Level** | **Controls (n=750)^a^** | **Cases (n=504)^b^** | **Total (n=1254)** | **p-value^c^** |
| --- | --- | --- | --- | --- | --- |
| **Age at Ascertainment** | Mean (SD) | 51.3 (16.9) | 46 (13.4) | 49.2 (15.8) | 2.81e-09 |
| **Sex** | Male | 398 (53.1%)^d^ | 238 (47.2%) | 636 (50.7%) | 0.05 |
|  | Female | 352 (46.9%) | 266 (52.8%) | 618 (49.3%) |  |
| **Diagnosis** | CP | 441 (58.8%) | 373 (74.0%) | 814 (64.9%) | 4.44e-08 |
|  | RAP | 309 (41.2%) | 131 (26.0%) | 440 (35.1%) |  |
| **BMI^e^** | Mean (SD) | 26 (6.1) | 25.7 (6) | 25.9 (6.1) | 0.35 |
| **Mental QOL^f^** | Mean (SD) | 47.3 (10.6) | 38.8 (11.7) | 43.7 (11.8) | 9.17e-37 |
|  | Missing | 96 | 24 | 120 |  |
| ^a^Patients without pain | | | | | |
| ^b^Patients with pain | | | | | |
| ^c^Pearson chi-squared for categorical; t test for continuous; two-tailed p < 0.05 considered significant | | | | | |
| ^d^Percentages shown next to counts are column percentages within each variable | | | | | |
| ^e^Body Mass Index | | | | | |
| ^f^Quality of Life | | | | | |

Table S2. Demographics of Constant-Severe Pain

| **Variable** | **Level** | **Controls (n=804)^a^** | **Cases (n=450)^b^** | **Total (n=1254)** | **p-value^c^** |
| --- | --- | --- | --- | --- | --- |
| **Age at Ascertainment** | Mean (SD) | 51 (16.7) | 46 (13.5) | 49.2 (15.8) | 8.08e-08 |
| **Sex** | Male | 426 (53.0%)^d^ | 210 (46.7%) | 636 (50.7%) | 0.04 |
|  | Female | 378 (47.0%) | 240 (53.3%) | 618 (49.3%) |  |
| **Diagnosis** | CP | 486 (60.4%) | 328 (72.9%) | 814 (64.9%) | 1.26e-05 |
|  | RAP | 318 (39.6%) | 122 (27.1%) | 440 (35.1%) |  |
| **BMI^e^** | Mean (SD) | 26 (6.1) | 25.7 (6.1) | 25.9 (6.1) | 0.37 |
| **Mental QOL^f^** | Mean (SD) | 46.6 (10.9) | 39 (11.8) | 43.7 (11.8) | 5.37e-28 |
|  | Missing | 101 | 19 | 120 |  |
| ^a^Patients without pain | | | | | |
| ^b^Patients with pain | | | | | |
| ^c^Pearson chi-squared for categorical; t test for continuous; two-tailed p < 0.05 considered significant | | | | | |
| ^d^Percentages shown next to counts are column percentages within each variable | | | | | |
| ^e^Body Mass Index | | | | | |
| ^f^Quality of Life | | | | | |

Table S3. Demographics of Severe Pain

| **Variable** | **Level** | **Controls (n=527)^a^** | **Cases (n=727)^b^** | **Total (n=1254)** | **p-value^c^** |
| --- | --- | --- | --- | --- | --- |
| **Age at Ascertainment** | Mean (SD) | 52.1 (16.1) | 47.1 (15.2) | 49.2 (15.8) | 1.68e-08 |
| **Sex** | Male | 262 (49.7%)^d^ | 374 (51.4%) | 636 (50.7%) | 0.58 |
|  | Female | 265 (50.3%) | 353 (48.6%) | 618 (49.3%) |  |
| **Diagnosis** | CP | 311 (59.0%) | 503 (69.2%) | 814 (64.9%) | 2.46e-04 |
|  | RAP | 216 (41.0%) | 224 (30.8%) | 440 (35.1%) |  |
| **BMI^e^** | Mean (SD) | 26.1 (6.2) | 25.7 (6) | 25.9 (6.1) | 0.29 |
| **Mental QOL^f^** | Mean (SD) | 46.7 (11) | 41.8 (12) | 43.7 (11.8) | 4.03e-12 |
|  | Missing | 88 | 32 | 120 |  |
| ^a^Patients without pain | | | | | |
| ^b^Patients with pain | | | | | |
| ^c^Pearson chi-squared for categorical; t test for continuous; two-tailed p < 0.05 considered significant | | | | | |
| ^d^Percentages shown next to counts are column percentages within each variable | | | | | |
| ^e^Body Mass Index | | | | | |
| ^f^Quality of Life | | | | | |


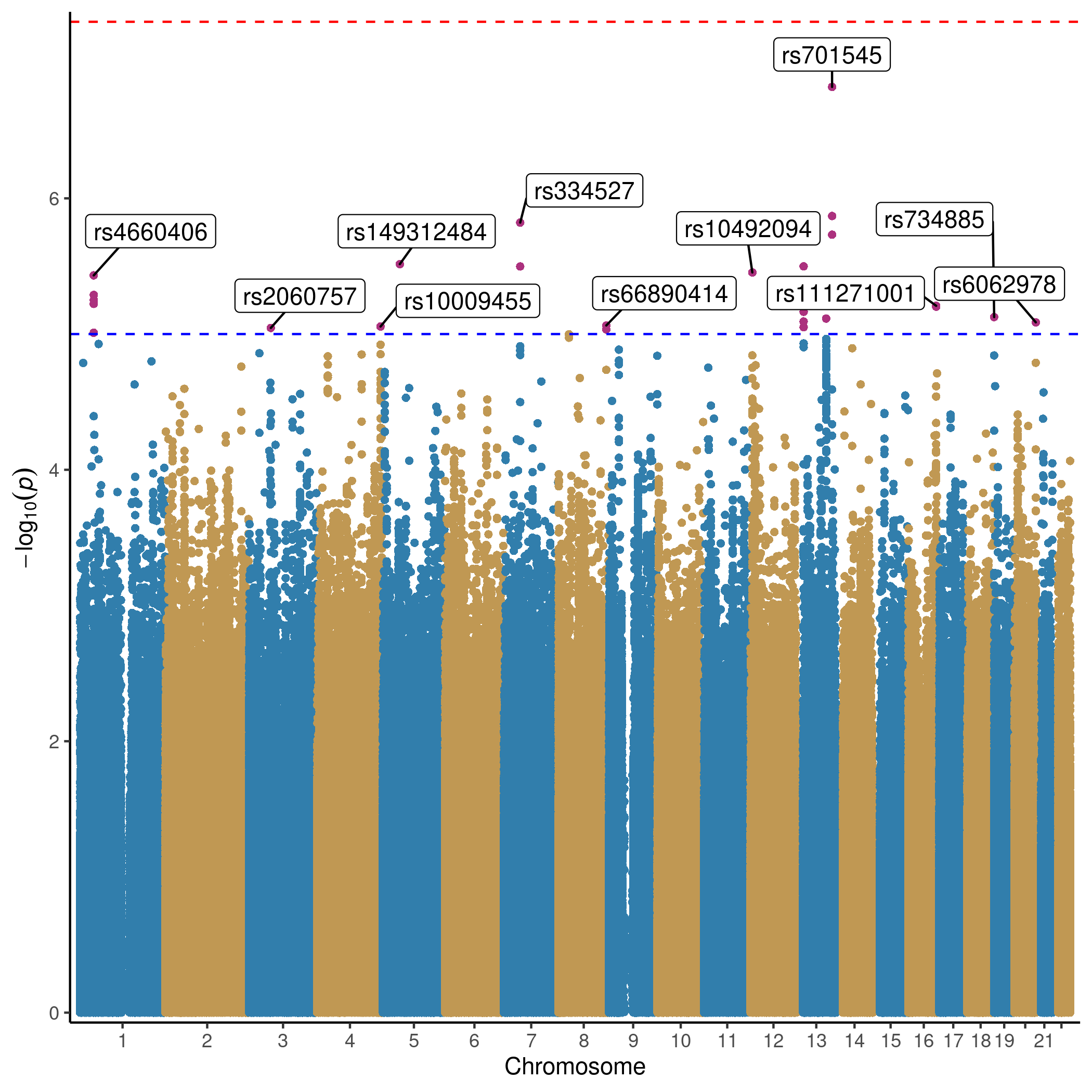


Figure S1. Manhattan Plot for Constant Pain


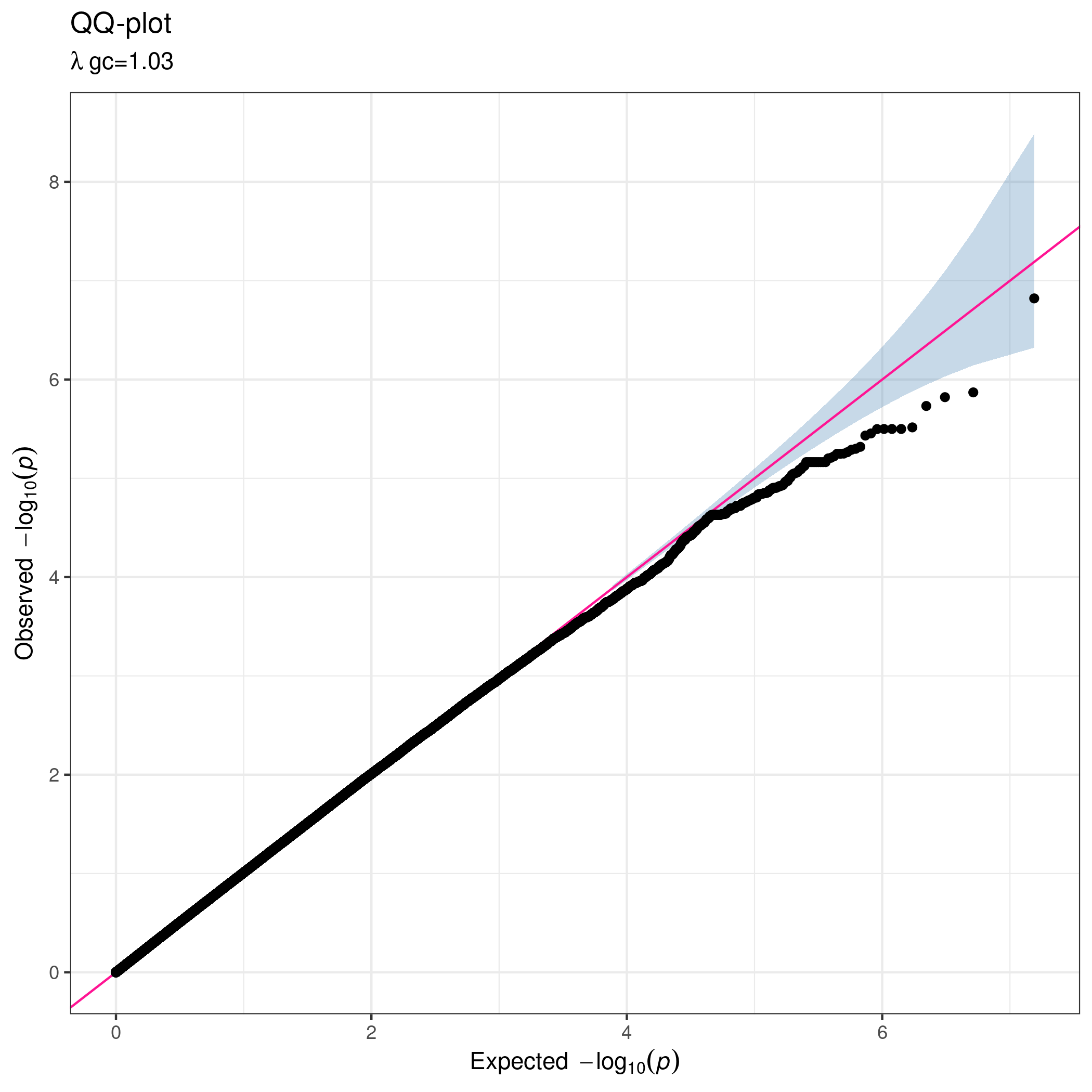


Figure S2 . QQ plot for Constant Pain

Table S4 . SNPs Meeting Suggestive Significance (1e-5) for Constant Pain

| **CHR** | **BP^1^** | **SNP^2^** | **OR** | **L95^3^** | **U95^4^** | **SE** | **P** | **A1^5^** | **A2^6^** | **MAF^7^** |
| --- | --- | --- | --- | --- | --- | --- | --- | --- | --- | --- |
| 1 | 40,814,528 | rs7522271 | 1.52 | 1.269 | 1.821 | 0.0922 | 5.62e-06 | C | T | 0.347 |
| 1 | 40,815,975 | rs2036197 | 1.505 | 1.261 | 1.796 | 0.0903 | 5.99e-06 | G | T | 0.368 |
| 1 | 40,819,094 | rs4660162 | 1.491 | 1.249 | 1.78 | 0.0903 | 9.78e-06 | G | A | 0.366 |
| 1 | 40,822,421 | rs4660405 | 1.511 | 1.265 | 1.804 | 0.0905 | 5.15e-06 | C | T | 0.367 |
| 1 | 40,823,404 | rs4660406 | 1.52 | 1.273 | 1.814 | 0.0904 | 3.69e-06 | C | T | 0.368 |
| 3 | 63,455,599 | rs2060757 | 0.6704 | 0.5619 | 0.7999 | 0.0901 | 9.01e-06 | T | C | 0.317 |
| 4 | 184,494,883 | rs10009455 | 0.453 | 0.3195 | 0.6423 | 0.178 | 8.80e-06 | G | A | 0.0466 |
| 5 | 49,435,222 | rs149312484 | 0.6283 | 0.5169 | 0.7637 | 0.0996 | 3.05e-06 | A | G | 0.205 |
| 7 | 47,565,793 | rs734899 | 0.6631 | 0.5578 | 0.7882 | 0.0882 | 3.18e-06 | G | A | 0.375 |
| 7 | 47,567,227 | rs334527 | 0.649 | 0.5442 | 0.774 | 0.0899 | 1.51e-06 | C | T | 0.326 |
| 7 | 47,571,488 | 7:47571488[A,C] | 0.6729 | 0.5678 | 0.7974 | 0.0866 | 4.80e-06 | A | C | 0.373 |
| 8 | 138,803,133 | rs36014323 | 0.5803 | 0.4562 | 0.7381 | 0.123 | 9.24e-06 | G | A | 0.123 |
| 8 | 138,803,658 | rs66890414 | 0.5786 | 0.4546 | 0.7363 | 0.123 | 8.64e-06 | T | C | 0.122 |
| 12 | 5,478,148 | rs10492094 | 0.6594 | 0.553 | 0.7862 | 0.0898 | 3.52e-06 | T | G | 0.282 |
| 13 | 20,850,871 | rs945371 | 0.5864 | 0.4638 | 0.7413 | 0.12 | 8.09e-06 | G | C | 0.125 |
| 13 | 20,853,876 | rs7327840 | 0.5876 | 0.466 | 0.7408 | 0.118 | 6.85e-06 | C | G | 0.13 |
| 13 | 20,853,886 | rs7326549 | 0.5876 | 0.466 | 0.7408 | 0.118 | 6.85e-06 | G | A | 0.13 |
| 13 | 20,853,986 | rs7333853 | 0.5876 | 0.466 | 0.7408 | 0.118 | 6.85e-06 | T | G | 0.13 |
| 13 | 20,854,161 | rs9552126 | 0.5876 | 0.466 | 0.7408 | 0.118 | 6.85e-06 | C | T | 0.13 |
| 13 | 20,854,191 | rs9552127 | 0.5876 | 0.466 | 0.7408 | 0.118 | 6.85e-06 | A | G | 0.13 |
| 13 | 20,854,299 | rs9552128 | 0.5876 | 0.466 | 0.7408 | 0.118 | 6.85e-06 | G | A | 0.13 |
| 13 | 20,854,384 | rs9552129 | 0.5876 | 0.466 | 0.7408 | 0.118 | 6.85e-06 | A | G | 0.13 |
| 13 | 20,854,763 | rs2050500 | 0.5846 | 0.4643 | 0.7362 | 0.118 | 5.03e-06 | A | G | 0.13 |
| 13 | 20,854,910 | 13:20854910[C,T] | 0.5777 | 0.4586 | 0.7277 | 0.118 | 3.17e-06 | C | T | 0.13 |
| 13 | 20,855,039 | rs2050498 | 0.5876 | 0.466 | 0.7408 | 0.118 | 6.85e-06 | T | C | 0.13 |
| 13 | 20,855,095 | rs4594101 | 0.5876 | 0.466 | 0.7408 | 0.118 | 6.85e-06 | C | T | 0.13 |
| 13 | 20,855,131 | rs2050497 | 0.5876 | 0.466 | 0.7408 | 0.118 | 6.85e-06 | A | G | 0.13 |
| 13 | 20,855,428 | rs9552130 | 0.5777 | 0.4586 | 0.7277 | 0.118 | 3.17e-06 | C | T | 0.13 |
| 13 | 20,855,444 | 13:20855444[C,T] | 0.5777 | 0.4586 | 0.7277 | 0.118 | 3.17e-06 | C | T | 0.13 |
| 13 | 20,857,149 | rs7329495 | 0.5836 | 0.4627 | 0.7361 | 0.118 | 5.44e-06 | C | G | 0.129 |
| 13 | 20,866,631 | rs2152451 | 0.5921 | 0.4698 | 0.7461 | 0.118 | 8.90e-06 | C | G | 0.131 |
| 13 | 20,866,838 | rs2152446 | 0.585 | 0.4641 | 0.7374 | 0.118 | 5.65e-06 | A | C | 0.131 |
| 13 | 20,866,839 | rs2152445 | 0.585 | 0.4641 | 0.7374 | 0.118 | 5.65e-06 | C | G | 0.131 |
| 13 | 86,362,179 | rs117027346 | 0.2706 | 0.1526 | 0.4798 | 0.292 | 7.69e-06 | T | C | 0.0149 |
| 13 | 103,580,361 | 13:103580361[A,G] | 0.6597 | 0.5561 | 0.7827 | 0.0872 | 1.85e-06 | G | A | 0.357 |
| 13 | 103,580,541 | rs701545 | 0.6397 | 0.5415 | 0.7558 | 0.0851 | 1.51e-07 | C | G | 0.395 |
| 13 | 103,606,829 | rs766223 | 1.503 | 1.274 | 1.773 | 0.0843 | 1.35e-06 | G | A | 0.529 |
| 16 | 81,259,428 | rs111271001 | 0.559 | 0.4344 | 0.7194 | 0.129 | 6.19e-06 | G | A | 0.106 |
| 16 | 81,260,855 | rs113101650 | 0.5571 | 0.4322 | 0.7181 | 0.13 | 6.29e-06 | T | G | 0.103 |
| 19 | 295,231 | rs734885 | 1.927 | 1.446 | 2.567 | 0.146 | 7.50e-06 | A | G | 0.12 |
| 20 | 62,256,590 | rs6062978 | 1.618 | 1.31 | 1.999 | 0.108 | 8.20e-06 | A | G | 0.226 |
| ^1^hg19 Base Pair | | | | | | | | | | |
| ^2^Blue SNPs were Identified as Lead SNPs by FUMA | | | | | | | | | | |
| ^3^Lower 95% Confidence Interval | | | | | | | | | | |
| ^4^Upper 95% Confidence Interval | | | | | | | | | | |
| ^5^Minor Allele | | | | | | | | | | |
| ^6^Major Allele | | | | | | | | | | |
| ^7^Minor Allele Frequency | | | | | | | | | | |


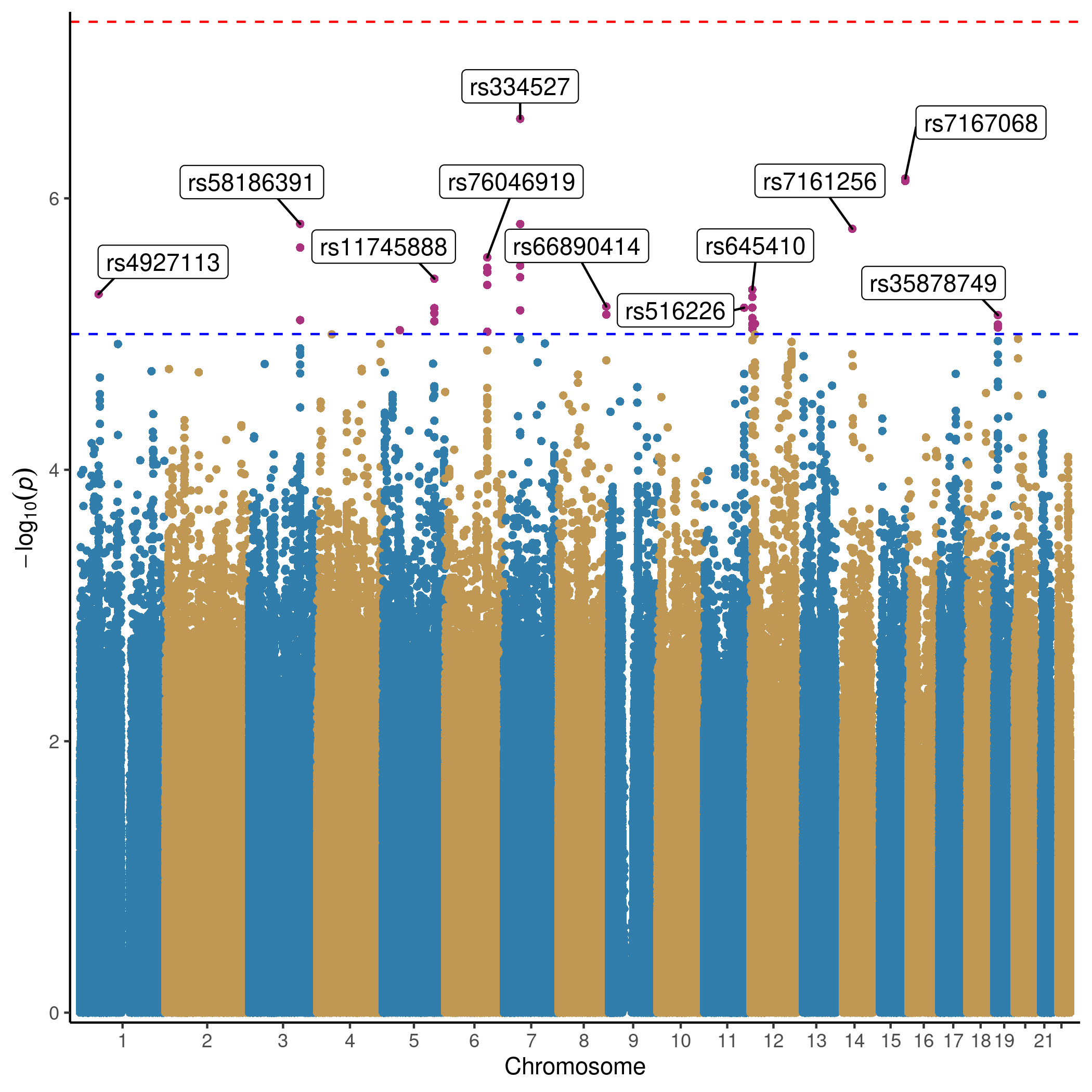


**Figure S3 .** Manhattan Plot for Constant-Severe Pain


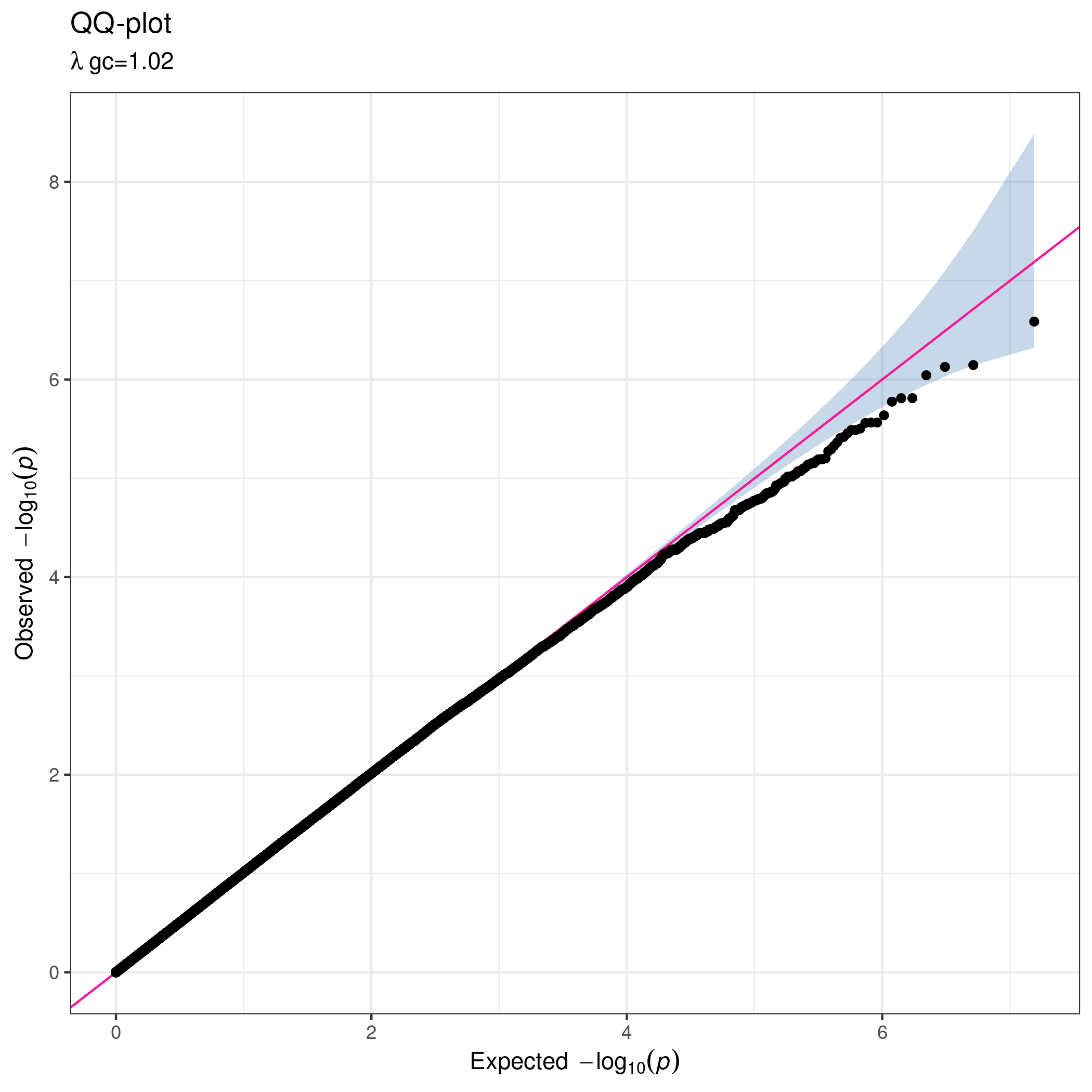


Figure S4 QQ Plot for Constant-Severe Pain

Table S5. SNPs Meeting Suggestive Significance (1e-5) for Constant-Severe Pain

| **CHR** | **BP^1^** | **SNP^2^** | **OR** | **L95^3^** | **U95^4^** | **SE** | **P** | **A1^5^** | **A2^6^** | **MAF^7^** |
| --- | --- | --- | --- | --- | --- | --- | --- | --- | --- | --- |
| 1 | 54,902,861 | rs4927113 | 0.6421 | 0.5308 | 0.7768 | 0.0971 | 5.08e-06 | C | T | 0.253 |
| 3 | 148,837,122 | rs772805 | 1.481 | 1.247 | 1.76 | 0.0879 | 7.89e-06 | A | G | 0.408 |
| 3 | 148,839,366 | rs58186391 | 1.547 | 1.295 | 1.848 | 0.0907 | 1.54e-06 | T | A | 0.383 |
| 3 | 148,848,923 | rs78985718 | 1.538 | 1.287 | 1.839 | 0.0912 | 2.30e-06 | G | A | 0.381 |
| 5 | 49,435,222 | rs149312484 | 0.6356 | 0.5201 | 0.7766 | 0.102 | 9.35e-06 | A | G | 0.203 |
| 5 | 149,965,575 | rs10058260 | 1.492 | 1.253 | 1.776 | 0.089 | 7.02e-06 | G | A | 0.486 |
| 5 | 149,966,193 | rs1363181 | 1.496 | 1.256 | 1.781 | 0.0892 | 6.44e-06 | A | G | 0.483 |
| 5 | 149,966,690 | rs10044420 | 1.488 | 1.25 | 1.771 | 0.089 | 8.04e-06 | A | G | 0.484 |
| 5 | 149,967,587 | rs6579794 | 1.492 | 1.253 | 1.776 | 0.089 | 7.02e-06 | G | A | 0.486 |
| 5 | 149,968,929 | rs11745888 | 1.512 | 1.268 | 1.802 | 0.0895 | 3.91e-06 | T | C | 0.483 |
| 6 | 122,878,836 | rs197687 | 1.48 | 1.244 | 1.761 | 0.0886 | 9.60e-06 | A | G | 0.392 |
| 6 | 122,885,419 | rs9388096 | 1.48 | 1.244 | 1.761 | 0.0886 | 9.60e-06 | C | T | 0.392 |
| 6 | 122,885,461 | rs9388097 | 1.48 | 1.244 | 1.761 | 0.0886 | 9.60e-06 | C | T | 0.392 |
| 6 | 122,897,790 | rs9482255 | 1.48 | 1.244 | 1.761 | 0.0886 | 9.60e-06 | G | A | 0.392 |
| 6 | 122,903,206 | rs76046919 | 0.4441 | 0.3163 | 0.6234 | 0.173 | 2.73e-06 | C | T | 0.0522 |
| 6 | 122,906,639 | rs56304640 | 0.4509 | 0.3221 | 0.6313 | 0.172 | 3.50e-06 | A | G | 0.0533 |
| 6 | 122,908,557 | rs9490487 | 0.4441 | 0.3163 | 0.6234 | 0.173 | 2.73e-06 | C | A | 0.0522 |
| 6 | 122,918,372 | rs4554346 | 0.45 | 0.3201 | 0.6327 | 0.174 | 4.35e-06 | A | G | 0.0522 |
| 6 | 122,919,311 | rs2082196 | 0.4451 | 0.3166 | 0.6259 | 0.174 | 3.24e-06 | C | G | 0.0522 |
| 6 | 122,921,183 | rs75083052 | 0.4451 | 0.3166 | 0.6259 | 0.174 | 3.24e-06 | G | T | 0.0522 |
| 7 | 47,565,793 | rs734899 | 0.6479 | 0.5428 | 0.7734 | 0.0903 | 1.55e-06 | G | A | 0.368 |
| 7 | 47,566,276 | rs334525 | 0.6507 | 0.5423 | 0.7808 | 0.093 | 3.81e-06 | C | T | 0.314 |
| 7 | 47,567,227 | rs334527 | 0.6212 | 0.5182 | 0.7446 | 0.0924 | 2.59e-07 | C | T | 0.317 |
| 7 | 47,569,436 | rs10265512 | 0.653 | 0.5464 | 0.7803 | 0.0909 | 2.75e-06 | G | A | 0.331 |
| 7 | 47,571,370 | rs7786087 | 0.6628 | 0.5576 | 0.7879 | 0.0882 | 3.13e-06 | A | G | 0.381 |
| 7 | 47,571,488 | 7:47571488[A,C] | 0.6462 | 0.5429 | 0.7692 | 0.0889 | 9.06e-07 | A | C | 0.363 |
| 7 | 47,573,637 | rs6964063 | 0.6585 | 0.549 | 0.7898 | 0.0928 | 6.69e-06 | A | C | 0.298 |
| 8 | 138,803,133 | rs36014323 | 0.5647 | 0.44 | 0.7248 | 0.127 | 7.19e-06 | G | A | 0.119 |
| 8 | 138,803,658 | rs66890414 | 0.5618 | 0.4374 | 0.7215 | 0.128 | 6.27e-06 | T | C | 0.118 |
| 11 | 116,519,655 | rs516226 | 1.65 | 1.327 | 2.05 | 0.111 | 6.39e-06 | T | C | 0.218 |
| 12 | 5,289,170 | rs7314052 | 1.584 | 1.299 | 1.931 | 0.101 | 5.33e-06 | C | A | 0.276 |
| 12 | 5,290,113 | rs12316588 | 1.578 | 1.294 | 1.923 | 0.101 | 6.38e-06 | T | C | 0.276 |
| 12 | 5,290,114 | rs12296611 | 1.565 | 1.284 | 1.908 | 0.101 | 9.14e-06 | G | A | 0.276 |
| 12 | 5,294,347 | rs2291095 | 1.566 | 1.286 | 1.908 | 0.101 | 8.48e-06 | T | G | 0.278 |
| 12 | 5,294,806 | rs2291094 | 1.57 | 1.288 | 1.912 | 0.101 | 7.61e-06 | T | C | 0.279 |
| 12 | 5,301,847 | rs645410 | 1.581 | 1.299 | 1.923 | 0.1 | 4.71e-06 | C | T | 0.283 |
| 12 | 12,990,341 | rs17394079 | 0.6038 | 0.4836 | 0.7539 | 0.113 | 8.41e-06 | T | C | 0.156 |
| 14 | 46,976,743 | rs7161256 | 2.035 | 1.521 | 2.721 | 0.148 | 1.68e-06 | A | G | 0.121 |
| 15 | 93,892,942 | rs7164857 | 0.6465 | 0.5439 | 0.7684 | 0.0882 | 7.46e-07 | T | G | 0.389 |
| 15 | 93,893,035 | rs7167068 | 0.646 | 0.5435 | 0.7678 | 0.0882 | 7.14e-07 | T | A | 0.393 |
| 19 | 11,229,765 | rs35878749 | 0.6654 | 0.5569 | 0.795 | 0.0908 | 7.26e-06 | A | G | 0.289 |
| 19 | 11,229,850 | rs34444274 | 0.6682 | 0.5593 | 0.7984 | 0.0908 | 8.97e-06 | G | C | 0.29 |
| 19 | 11,230,402 | rs12611067 | 0.6678 | 0.5591 | 0.7977 | 0.0907 | 8.51e-06 | T | G | 0.29 |
| ^1^hg19 Base Pair | | | | | | | | | | |
| ^2^Blue SNPs were Identified as Lead SNPs by FUMA | | | | | | | | | | |
| ^3^Lower 95% Confidence Interval | | | | | | | | | | |
| ^4^Upper 95% Confidence Interval | | | | | | | | | | |
| ^5^Minor Allele | | | | | | | | | | |
| ^6^Major Allele | | | | | | | | | | |
| ^7^Minor Allele Frequency | | | | | | | | | | |


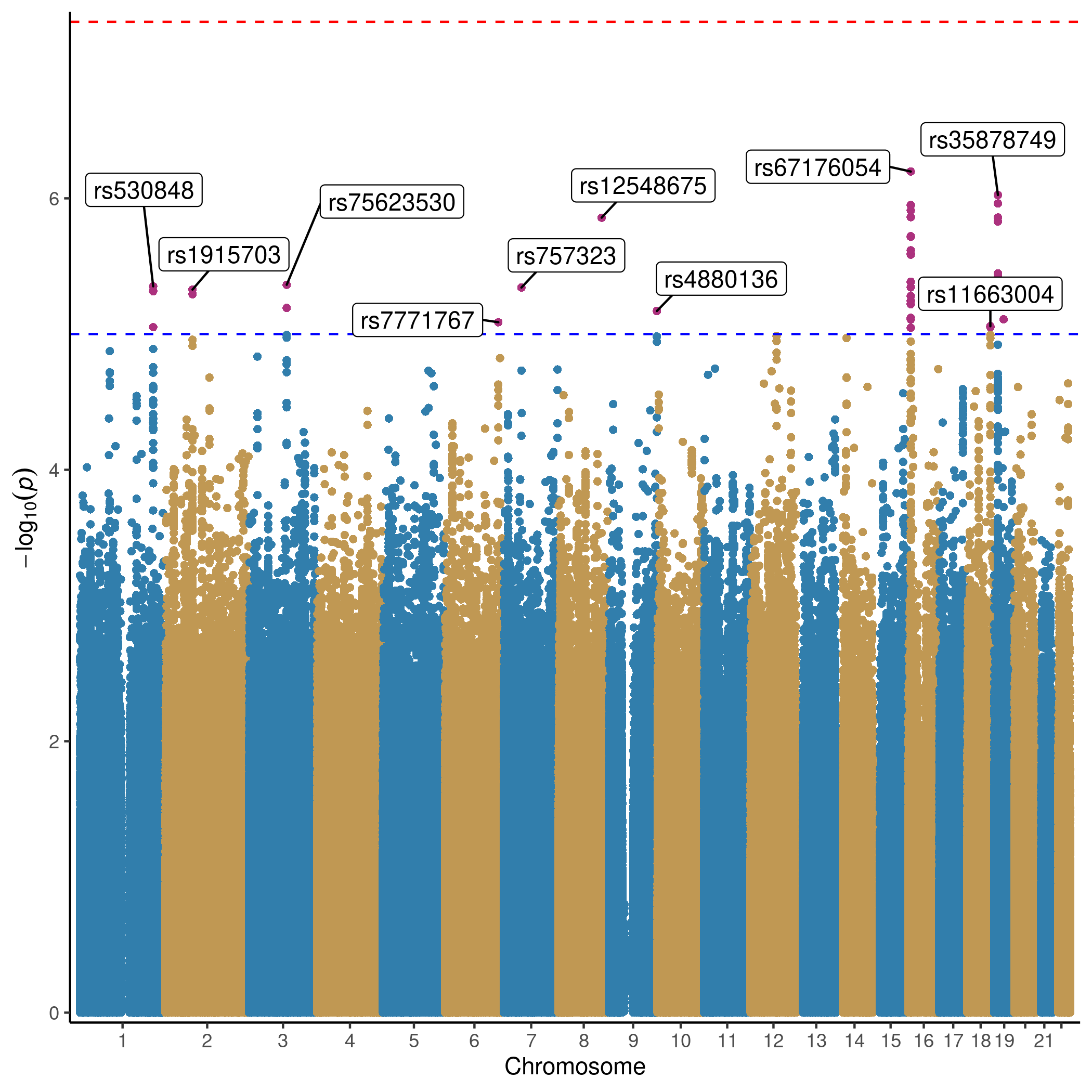


Figure S5. Manhattan Plot for Severe Pain


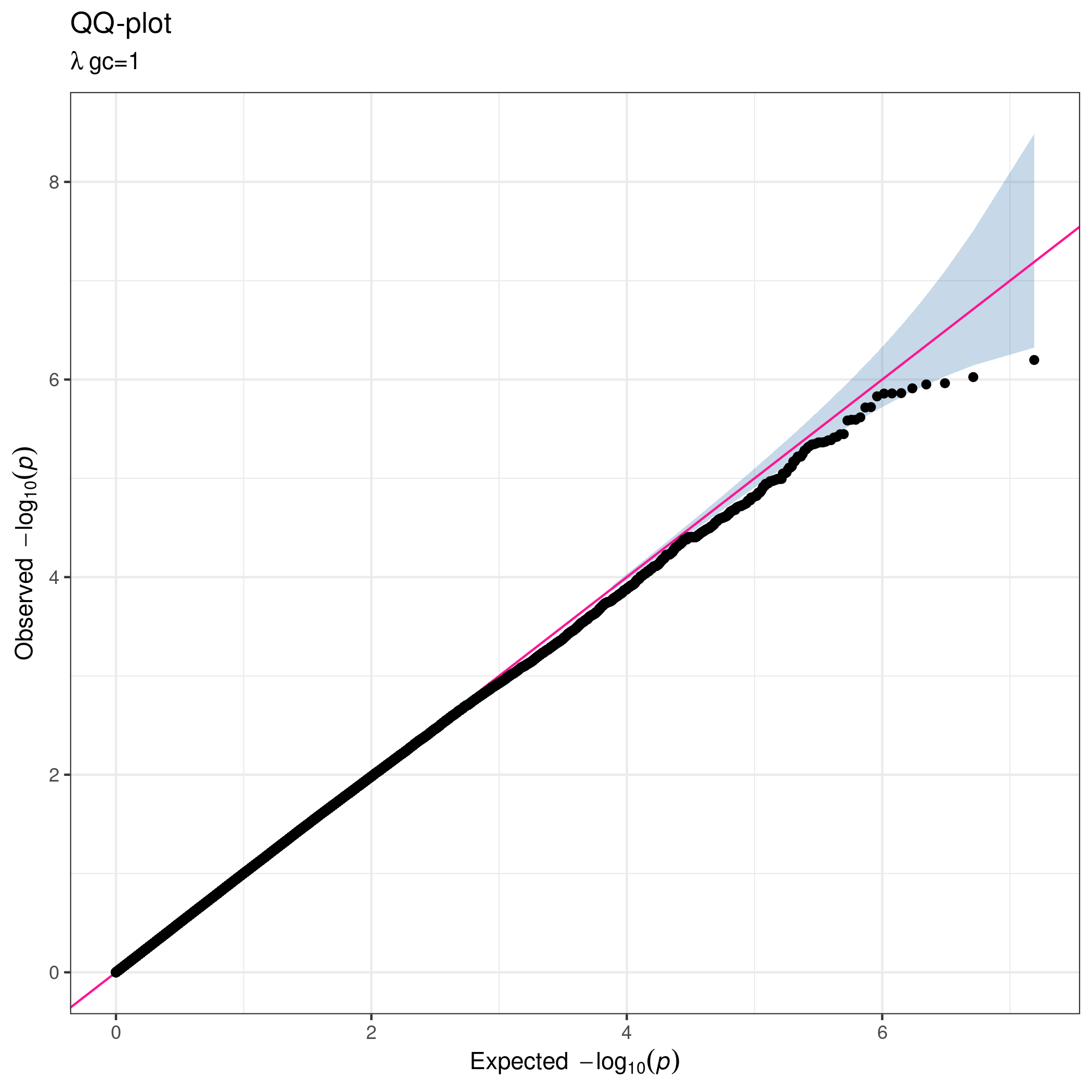


Figure S6 *.* QQ plot for Severe Pain

Table S6 *.* SNPs Meeting Suggestive Significance (1e-5) for Severe Pain

| **CHR** | **BP^1^** | **SNP^2^** | **OR** | **L95^3^** | **U95^4^** | **SE** | **P** | **A1^5^** | **A2^6^** | **MAF^7^** |
| --- | --- | --- | --- | --- | --- | --- | --- | --- | --- | --- |
| 1 | 213,732,037 | rs431573 | 0.606 | 0.4858 | 0.7558 | 0.113 | 8.90e-06 | A | G | 0.144 |
| 1 | 213,732,214 | rs530848 | 0.6072 | 0.4907 | 0.7514 | 0.109 | 4.45e-06 | C | G | 0.155 |
| 1 | 213,749,065 | rs6682832 | 0.5843 | 0.4641 | 0.7357 | 0.117 | 4.84e-06 | C | T | 0.129 |
| 2 | 78,830,744 | rs1239078 | 1.525 | 1.272 | 1.828 | 0.0925 | 5.08e-06 | G | A | 0.325 |
| 2 | 78,832,777 | rs1915703 | 1.527 | 1.274 | 1.83 | 0.0924 | 4.70e-06 | A | G | 0.325 |
| 3 | 109,644,774 | rs59868665 | 0.1839 | 0.0881 | 0.3837 | 0.375 | 6.41e-06 | C | A | 0.00688 |
| 3 | 109,672,395 | rs75623530 | 0.1795 | 0.0863 | 0.3735 | 0.374 | 4.33e-06 | A | G | 0.00688 |
| 3 | 109,673,273 | rs116440411 | 0.1795 | 0.0863 | 0.3735 | 0.374 | 4.33e-06 | T | C | 0.00688 |
| 3 | 109,675,891 | rs140099063 | 0.1795 | 0.0863 | 0.3735 | 0.374 | 4.33e-06 | G | C | 0.00688 |
| 6 | 155,038,479 | rs7771767 | 1.451 | 1.232 | 1.709 | 0.0834 | 8.18e-06 | A | G | 0.547 |
| 7 | 51,077,759 | rs757323 | 1.476 | 1.249 | 1.742 | 0.0848 | 4.54e-06 | G | A | 0.514 |
| 8 | 125,224,719 | rs12548675 | 0.6237 | 0.5149 | 0.7555 | 0.0978 | 1.39e-06 | T | C | 0.202 |
| 9 | 139,620,311 | rs4880136 | 0.6762 | 0.5703 | 0.8018 | 0.0869 | 6.76e-06 | A | G | 0.447 |
| 9 | 139,621,168 | rs2275160 | 0.6762 | 0.5703 | 0.8018 | 0.0869 | 6.76e-06 | G | A | 0.447 |
| 16 | 7,362,235 | rs28591292 | 2.241 | 1.601 | 3.137 | 0.172 | 2.60e-06 | C | T | 0.102 |
| 16 | 7,365,355 | 16:7365355[A,G] | 2.274 | 1.616 | 3.199 | 0.174 | 2.42e-06 | A | G | 0.0997 |
| 16 | 7,367,240 | rs12918524 | 2.169 | 1.554 | 3.027 | 0.17 | 5.24e-06 | C | A | 0.102 |
| 16 | 7,367,453 | rs34265202 | 2.187 | 1.568 | 3.052 | 0.17 | 4.11e-06 | G | A | 0.102 |
| 16 | 7,368,357 | rs11864520 | 2.073 | 1.52 | 2.828 | 0.158 | 4.13e-06 | G | A | 0.115 |
| 16 | 7,369,787 | rs35999696 | 2.012 | 1.481 | 2.732 | 0.156 | 7.59e-06 | G | C | 0.116 |
| 16 | 7,369,880 | rs35767895 | 2.041 | 1.498 | 2.78 | 0.158 | 6.03e-06 | G | A | 0.115 |
| 16 | 7,370,244 | rs55870430 | 2.041 | 1.498 | 2.78 | 0.158 | 6.03e-06 | C | T | 0.115 |
| 16 | 7,370,418 | rs36095768 | 2.041 | 1.498 | 2.78 | 0.158 | 6.03e-06 | G | T | 0.115 |
| 16 | 7,370,953 | rs8052441 | 2.06 | 1.513 | 2.805 | 0.158 | 4.52e-06 | G | C | 0.116 |
| 16 | 7,371,066 | rs67176054 | 2.585 | 1.779 | 3.757 | 0.191 | 6.34e-07 | A | G | 0.0894 |
| 16 | 7,380,549 | rs34109083 | 2.371 | 1.675 | 3.356 | 0.177 | 1.12e-06 | G | A | 0.099 |
| 16 | 7,381,299 | rs17739067 | 2.329 | 1.645 | 3.297 | 0.177 | 1.90e-06 | G | A | 0.0977 |
| 16 | 7,383,759 | rs67729500 | 2.257 | 1.608 | 3.168 | 0.173 | 2.56e-06 | T | A | 0.1 |
| 16 | 7,384,352 | rs4362406 | 2.298 | 1.632 | 3.236 | 0.175 | 1.92e-06 | A | G | 0.0997 |
| 16 | 7,384,503 | rs4516245 | 2.257 | 1.608 | 3.168 | 0.173 | 2.56e-06 | C | G | 0.1 |
| 16 | 7,385,103 | rs17143464 | 2.348 | 1.663 | 3.315 | 0.176 | 1.23e-06 | T | C | 0.0997 |
| 16 | 7,385,942 | rs34009260 | 2.355 | 1.663 | 3.334 | 0.177 | 1.37e-06 | A | G | 0.0984 |
| 16 | 7,396,323 | rs72769295 | 2.237 | 1.572 | 3.185 | 0.18 | 7.80e-06 | T | C | 0.0915 |
| 16 | 7,405,611 | rs7202500 | 2.263 | 1.59 | 3.22 | 0.18 | 5.67e-06 | T | C | 0.0922 |
| 16 | 7,417,955 | rs35508038 | 2.253 | 1.574 | 3.224 | 0.183 | 8.98e-06 | T | C | 0.0887 |
| 18 | 67,322,660 | rs11151522 | 0.6873 | 0.5824 | 0.8109 | 0.0844 | 8.91e-06 | A | G | 0.411 |
| 18 | 67,324,345 | rs11663004 | 0.6872 | 0.5825 | 0.8108 | 0.0844 | 8.75e-06 | A | G | 0.411 |
| 19 | 11,221,180 | rs892116 | 0.685 | 0.5835 | 0.8042 | 0.0818 | 3.80e-06 | A | G | 0.362 |
| 19 | 11,224,265 | rs5930 | 0.6863 | 0.5846 | 0.8058 | 0.0819 | 4.26e-06 | A | G | 0.358 |
| 19 | 11,227,070 | rs2738445 | 0.6863 | 0.5853 | 0.8047 | 0.0812 | 3.57e-06 | C | T | 0.382 |
| 19 | 11,227,480 | 19:11227480[A,C] | 0.6859 | 0.5848 | 0.8045 | 0.0813 | 3.58e-06 | A | C | 0.372 |
| 19 | 11,228,745 | rs2569550 | 0.6871 | 0.586 | 0.8058 | 0.0812 | 3.87e-06 | T | C | 0.369 |
| 19 | 11,229,765 | rs35878749 | 0.6576 | 0.5561 | 0.7775 | 0.0855 | 9.45e-07 | A | G | 0.307 |
| 19 | 11,229,850 | rs34444274 | 0.6591 | 0.5574 | 0.7794 | 0.0855 | 1.09e-06 | G | C | 0.308 |
| 19 | 11,230,362 | rs12609673 | 0.6649 | 0.5631 | 0.7851 | 0.0848 | 1.48e-06 | C | A | 0.321 |
| 19 | 11,230,402 | rs12611067 | 0.6622 | 0.5601 | 0.7828 | 0.0854 | 1.38e-06 | T | G | 0.309 |
| 19 | 27,992,394 | rs62111935 | 0.572 | 0.4478 | 0.7307 | 0.125 | 7.79e-06 | A | G | 0.105 |
| ^1^hg19 Base Pair | | | | | | | | | | |
| ^2^Blue SNPs were Identified as Lead SNPs by FUMA | | | | | | | | | | |
| ^3^Lower 95% Confidence Interval | | | | | | | | | | |
| ^4^Upper 95% Confidence Interval | | | | | | | | | | |
| ^5^Minor Allele | | | | | | | | | | |
| ^6^Major Allele | | | | | | | | | | |
| ^7^Minor Allele Frequency | | | | | | | | | | |

Table S7 *.* FUMA Genomic Loci for Constant Pain

| **GenomicLocusRegion^1^** | **rsID^2^** | **chr** | **pos** | **p^3^** | **nGWASSNPs^4^** | **nIndSigSNPs^5^** | **IndSigSNPs^6^** | **nearestGene** |
| --- | --- | --- | --- | --- | --- | --- | --- | --- |
| 1:40814528-40823404 | rs4660406 | 1 | 40,823,404 | 3.69e-06 | 5 | 1 | rs4660406 | *SMAP2* |
| 3:63441514-63455599 | rs2060757 | 3 | 63,455,599 | 9.01e-06 | 5 | 1 | rs2060757 | *SYNPR:SYNPR-AS1* |
| 4:184484425-184505515 | rs10009455 | 4 | 184,494,883 | 8.80e-06 | 16 | 1 | rs10009455 | *ING2* |
| 5:49435222-49435222 | rs149312484 | 5 | 49,435,222 | 3.05e-06 | 1 | 1 | rs149312484 | *EMB* |
| 7:47565793-47575970 | rs334527 | 7 | 47,567,227 | 1.51e-06 | 13 | 1 | rs334527 | *TNS3* |
| 8:138803133-138806916 | rs66890414 | 8 | 138,803,658 | 8.64e-06 | 3 | 1 | rs66890414 | *FAM135B* |
| 12:5441541-5487932 | rs10492094 | 12 | 5,478,148 | 3.52e-06 | 3 | 1 | rs10492094 | *NTF3* |
| 13:20847066-20866839 | 13:20855444[C,T] | 13 | 20,855,444 | 3.17e-06 | 35 | 1 | 13:20855444[C,T] | *GJB6* |
| 13:86362179-86565426 | rs117027346 | 13 | 86,362,179 | 7.69e-06 | 100 | 1 | rs117027346 | *SLITRK6* |
| 13:103580361-103606829 | rs701545 | 13 | 103,580,541 | 1.51e-07 | 3 | 1 | rs701545 | *METTL21EP* |
| 16:81238750-81264177 | rs111271001 | 16 | 81,259,428 | 6.19e-06 | 21 | 1 | rs111271001 | *PKD1L2* |
| 19:295231-295295 | rs734885 | 19 | 295,231 | 7.50e-06 | 2 | 1 | rs734885 | *PPAP2C* |
| 20:62200860-62263747 | rs6062978 | 20 | 62,256,590 | 8.20e-06 | 6 | 1 | rs6062978 | *GMEB2* |
| ^1^chr:start-end based on hg19; ^2^rsID of top lead SNP; ^3^GWAS p value; ^4^Number of unique GWAS SNPs in locus; ^5^Number of LD independent SNPs; ^6^rsID of independent SNPs | | | | | | | | |

Table S8 *.* FUMA Genomic Loci for Constant-Severe Pain

| **GenomicLocusRegion^1^** | **rsID^2^** | **chr** | **pos** | **p^3^** | **nGWASSNPs^4^** | **nIndSigSNPs^5^** | **IndSigSNPs^6^** | **nearestGene** |
| --- | --- | --- | --- | --- | --- | --- | --- | --- |
| 1:54896755-54922021 | rs4927113 | 1 | 54,902,861 | 5.08e-06 | 15 | 1 | rs4927113 | *SSBP3* |
| 3:148698474-148876261 | rs58186391 | 3 | 148,839,366 | 1.54e-06 | 53 | 1 | rs58186391 | *HPS3* |
| 5:49435222-49435222 | rs149312484 | 5 | 49,435,222 | 9.35e-06 | 1 | 1 | rs149312484 | *EMB* |
| 5:149954864-149990727 | rs11745888 | 5 | 149,968,929 | 3.91e-06 | 40 | 1 | rs11745888 | *SYNPO* |
| 6:122429305-122921183 | rs76046919 | 6 | 122,903,206 | 2.73e-06 | 103 | 2 | rs9388097;rs76046919 | *PKIB* |
| 7:47565793-47575970 | rs334527 | 7 | 47,567,227 | 2.59e-07 | 13 | 1 | rs334527 | *TNS3* |
| 8:138803133-138806916 | rs66890414 | 8 | 138,803,658 | 6.27e-06 | 3 | 1 | rs66890414 | *FAM135B* |
| 11:116519655-116519655 | rs516226 | 11 | 116,519,655 | 6.39e-06 | 1 | 1 | rs516226 | *AP000770.1* |
| 12:5284122-5315245 | rs645410 | 12 | 5,301,847 | 4.71e-06 | 17 | 1 | rs645410 | *RP11-319E16.1* |
| 12:12963744-12990341 | rs17394079 | 12 | 12,990,341 | 8.41e-06 | 10 | 1 | rs17394079 | *DDX47* |
| 14:46976743-46986881 | rs7161256 | 14 | 46,976,743 | 1.68e-06 | 2 | 1 | rs7161256 | *LINC00871* |
| 15:93892942-93908051 | rs7167068 | 15 | 93,893,035 | 7.14e-07 | 4 | 1 | rs7167068 | *RGMA* |
| 19:11221180-11232696 | rs35878749 | 19 | 11,229,765 | 7.26e-06 | 10 | 1 | rs35878749 | *LDLR* |
| ^1^chr:start-end based on hg19; ^2^rsID of top lead SNP; ^3^GWAS p value; ^4^Number of unique GWAS SNPs in locus; ^5^Number of LD independent SNPs; ^6^rsID of independent SNPs | | | | | | | | |

Table S9*.* FUMA Genomic Loci for Severe Pain

| **GenomicLocusRegion^1^** | **rsID^2^** | **chr** | **pos** | **p^3^** | **nGWASSNPs^4^** | **nIndSigSNPs^5^** | **IndSigSNPs^6^** | **nearestGene** |
| --- | --- | --- | --- | --- | --- | --- | --- | --- |
| 1:213685950-213755621 | rs530848 | 1 | 213,732,214 | 4.45e-06 | 27 | 1 | rs530848 | *RPL31P13* |
| 2:78764895-78837866 | rs1915703 | 2 | 78,832,777 | 4.70e-06 | 11 | 1 | rs1915703 | *CYCSP6* |
| 3:109525798-109681921 | rs75623530 | 3 | 109,672,395 | 4.33e-06 | 30 | 1 | rs75623530 | *MIR4445* |
| 6:155022713-155160128 | rs7771767 | 6 | 155,038,479 | 8.18e-06 | 76 | 1 | rs7771767 | *SCAF8* |
| 7:51035899-51079151 | rs757323 | 7 | 51,077,759 | 4.54e-06 | 6 | 1 | rs757323 | *COBL* |
| 8:125224719-125224719 | rs12548675 | 8 | 125,224,719 | 1.39e-06 | 1 | 1 | rs12548675 | *RP11-37N22.1* |
| 9:139614170-139642961 | rs2275160 | 9 | 139,621,168 | 6.76e-06 | 10 | 1 | rs2275160 | *SNHG7* |
| 16:7353976-7417955 | rs67176054 | 16 | 7,371,066 | 6.34e-07 | 54 | 2 | rs67176054;rs34109083 | *RBFOX1* |
| 18:67306031-67327598 | rs11663004 | 18 | 67,324,345 | 8.75e-06 | 26 | 1 | rs11663004 | *DOK6* |
| 19:11221180-11232696 | rs35878749 | 19 | 11,229,765 | 9.45e-07 | 10 | 1 | rs35878749 | *LDLR* |
| 19:27947716-28309577 | rs62111935 | 19 | 27,992,394 | 7.79e-06 | 93 | 1 | rs62111935 | *LINC00662* |
| ^1^chr:start-end based on hg19; ^2^rsID of top lead SNP; ^3^GWAS p value; ^4^Number of unique GWAS SNPs in locus; ^5^Number of LD independent SNPs; ^6^rsID of independent SNPs | | | | | | | | |

Table S10

S-MultiXcan Results for Constant Pain (see [Table S13](#A3ST13): Legend for S-MultiXcan Variables)

| **gene** | **gene_name** | **pvalue** | **n** | **n_indep** | **p_i_best** | **t_i_best** | **p_i_worst** | **t_i_worst** | **eigen_max** | **eigen_min** | **eigen_min_kept** | **z_min** | **z_max** | **z_mean** | **z_sd** |
| --- | --- | --- | --- | --- | --- | --- | --- | --- | --- | --- | --- | --- | --- | --- | --- |
| ENSG00000161021.11 | *MAML1* | 2.07e-07 | 44 | 4 | 3.39e-02 | Heart_Left_Ventricle | 0.75 | Spleen | 29.2 | 2.86e-15 | 1.44 | -2.12 | 1.81 | -1.14 | 1.19 |
| ENSG00000163121.9 | *NEURL3* | 9.28e-06 | 11 | 3 | 1.22e-03 | Brain_Substantia_nigra | 0.761 | Brain_Nucleus_accumbens_basal_ganglia | 9.36 | 3.55e-18 | 0.701 | -3.23 | 3.23 | 0.678 | 2.8 |
| ENSG00000162438.11 | *CTRC* | 2.45e-05 | 8 | 6 | 5.51e-04 | Pancreas | 0.358 | Testis | 3.72 | 2.78e-17 | 0.387 | -3.45 | 3.4 | 0.323 | 2.39 |

Table S11

S-MultiXcan Results for Constant-Severe Pain (see [Table S13](#A3ST13): Legend for S-MultiXcan Variables)

| **gene** | **gene_name** | **pvalue** | **n** | **n_indep** | **p_i_best** | **t_i_best** | **p_i_worst** | **t_i_worst** | **eigen_max** | **eigen_min** | **eigen_min_kept** | **z_min** | **z_max** | **z_mean** | **z_sd** |
| --- | --- | --- | --- | --- | --- | --- | --- | --- | --- | --- | --- | --- | --- | --- | --- |
| ENSG00000161021.11 | *MAML1* | 4.99e-08 | 44 | 4 | 6.19e-02 | Brain_Cerebellar_Hemisphere | 0.837 | Spleen | 29.2 | 2.86e-15 | 1.44 | -1.87 | 1.77 | -0.882 | 1.23 |
| ENSG00000025156.12 | *HSF2* | 5.85e-06 | 46 | 5 | 3.09e-05 | Skin_Not_Sun_Exposed_Suprapubic | 0.469 | Artery_Aorta | 29.8 | 1.01e-15 | 1.21 | -3.22 | 4.17 | 0.79 | 2.38 |
| ENSG00000162438.11 | *CTRC* | 4.5e-05 | 8 | 6 | 2.29e-03 | Pancreas | 0.316 | Whole_Blood | 3.72 | 2.78e-17 | 0.387 | -3.05 | 2.99 | 0.488 | 2.3 |
| ENSG00000151789.10 | *ZNF385D* | 8.25e-05 | 7 | 7 | 4.85e-03 | Artery_Aorta | 0.814 | Spleen | 1.74 | 0.439 | 0.439 | -2.82 | 2.61 | 0.456 | 1.7 |

Table S12

S-MultiXcan Results for Severe Pain (see [Table S13](#A3ST13): Legend for S-MultiXcan Variables)

| **gene** | **gene_name** | **pvalue** | **n** | **n_indep** | **p_i_best** | **t_i_best** | **p_i_worst** | **t_i_worst** | **eigen_max** | **eigen_min** | **eigen_min_kept** | **z_min** | **z_max** | **z_mean** | **z_sd** |
| --- | --- | --- | --- | --- | --- | --- | --- | --- | --- | --- | --- | --- | --- | --- | --- |
| ENSG00000130164.13 | *LDLR* | 6.53e-05 | 3 | 2 | 2.16e-05 | Artery_Tibial | 0.259 | Pancreas | 2.36 | 2.3e-17 | 0.643 | -4.25 | -1.13 | -2.17 | 1.8 |
| ENSG00000206052.10 | *DOK6* | 7.5e-05 | 14 | 10 | 8.75e-06 | Nerve_Tibial | 0.864 | Brain_Hypothalamus | 3.58 | 4.87e-17 | 0.137 | -4.45 | 2.25 | -0.554 | 1.84 |

Table S13*;* Legend for S-MultiXcan Variables

| gene: a gene's id. |
| --- |
| gene_name: gene name. |
| pvalue: p-value of S-MultiXcan association. |
| n: number of tissues available for gene. |
| n_indep: number of independent components of variation kept among the tissues' predictions. |
| p_i_best: best p-value of single-tissue S-PrediXcan association. |
| t_i_best: name of best single-tissue S-PrediXcan association. |
| p_i_worst: worst p-value of single-tissue S-PrediXcan association. |
| t_i_worst: name of worst single-tissue S-PrediXcan association. |
| eigen_max: In the SVD decomposition of predicted expression correlation matrix: eigenvalue (variance explained) of the top independent component. |
| eigen_min: In the SVD decomposition of predicted expression correlation matrix: eigenvalue (variance explained) of the last independent component. |
| eigen_min_kept: In the SVD decomposition of predicted expression correlation matrix: eigenvalue (variance explained) of the smallest independent component that was kept. |
| z_min: minimum z-score among single-tissue S-PrediXcan associations. |
| z_max: maximum z-score among single-tissue S-PrediXcan associations. |
| z_mean: mean z-score among single-tissue S-PrediXcan associations. |
| z_sd: standard deviation of the mean z-score among single-tissue S-PrediXcan associations. |


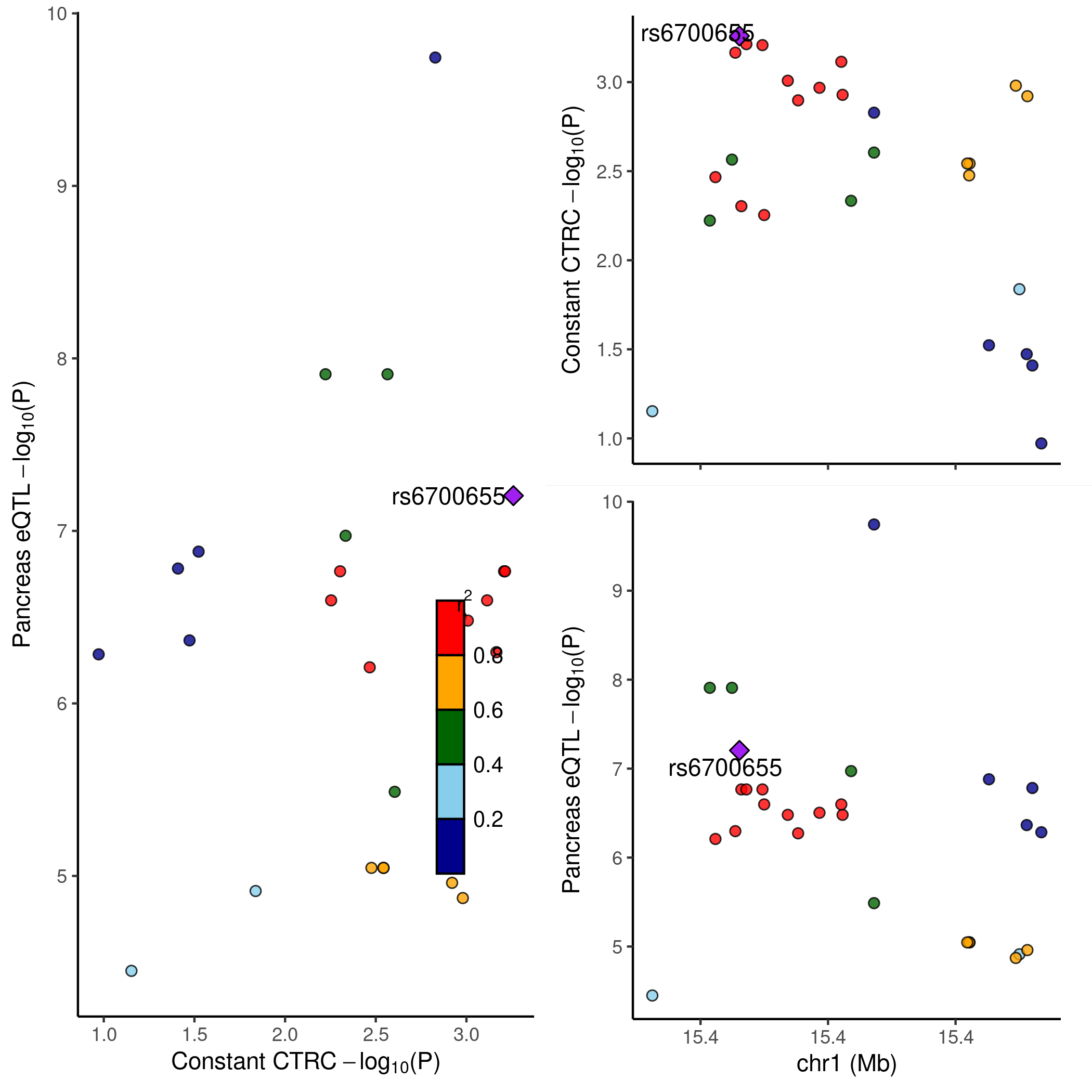


Figure S7

Locuscompare Plot for Constant Pain CTRC and Pancreas eQTL. Top right: regional scatter plot for GWAS. Bottom right: regional scatter plot for eQTL. Left: joint distribution of p-values from GWAS and eQTL. Color represents LD with lead SNP.


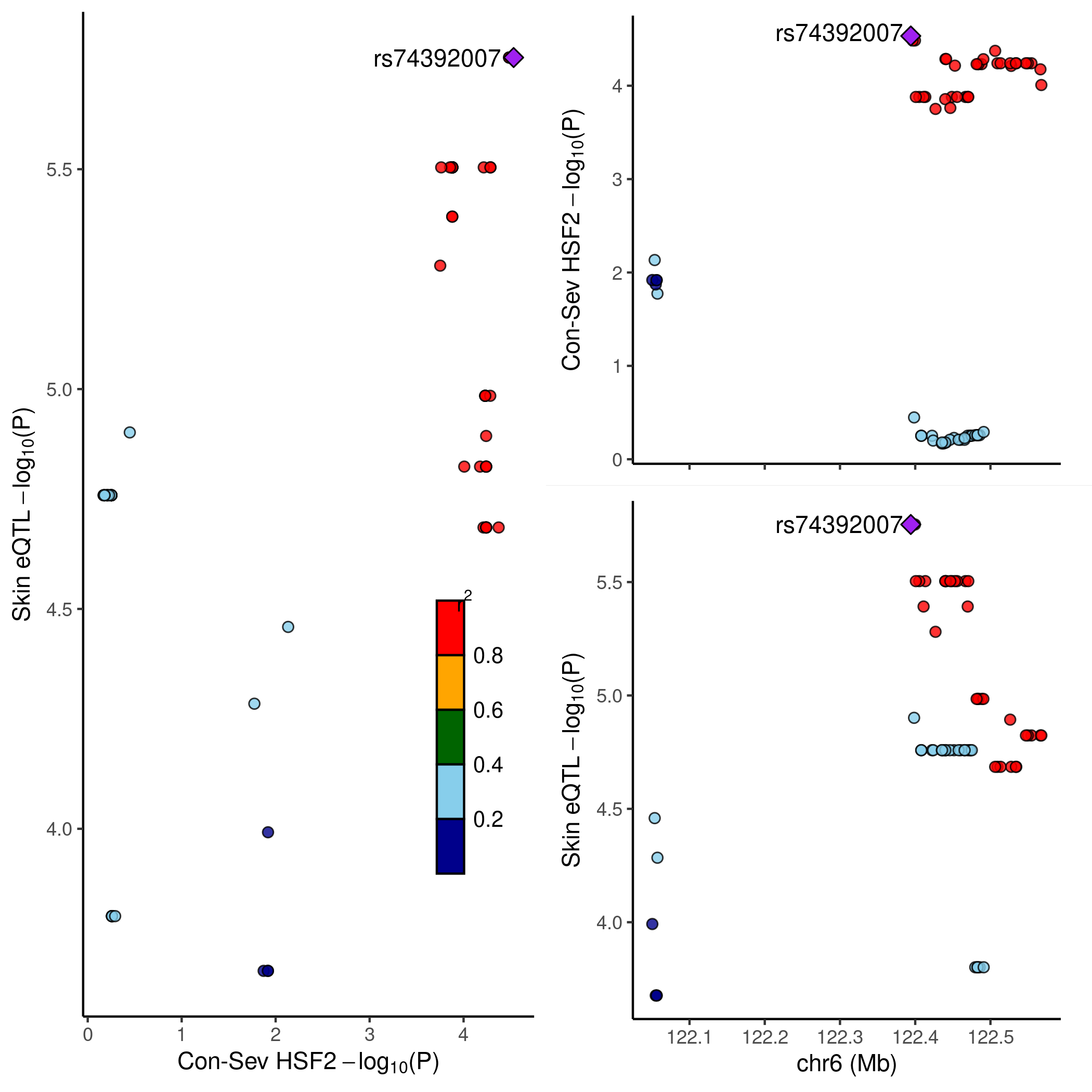


Figure S8

Locuscompare Plot for Constant-Severe Pain HSF2 and Skin Not Sun Exposed Suprapubic eQTL. Top right: regional scatter plot for GWAS. Bottom right: regional scatter plot for eQTL. Left: joint distribution of p-values from GWAS and eQTL. Color represents LD with lead SNP.


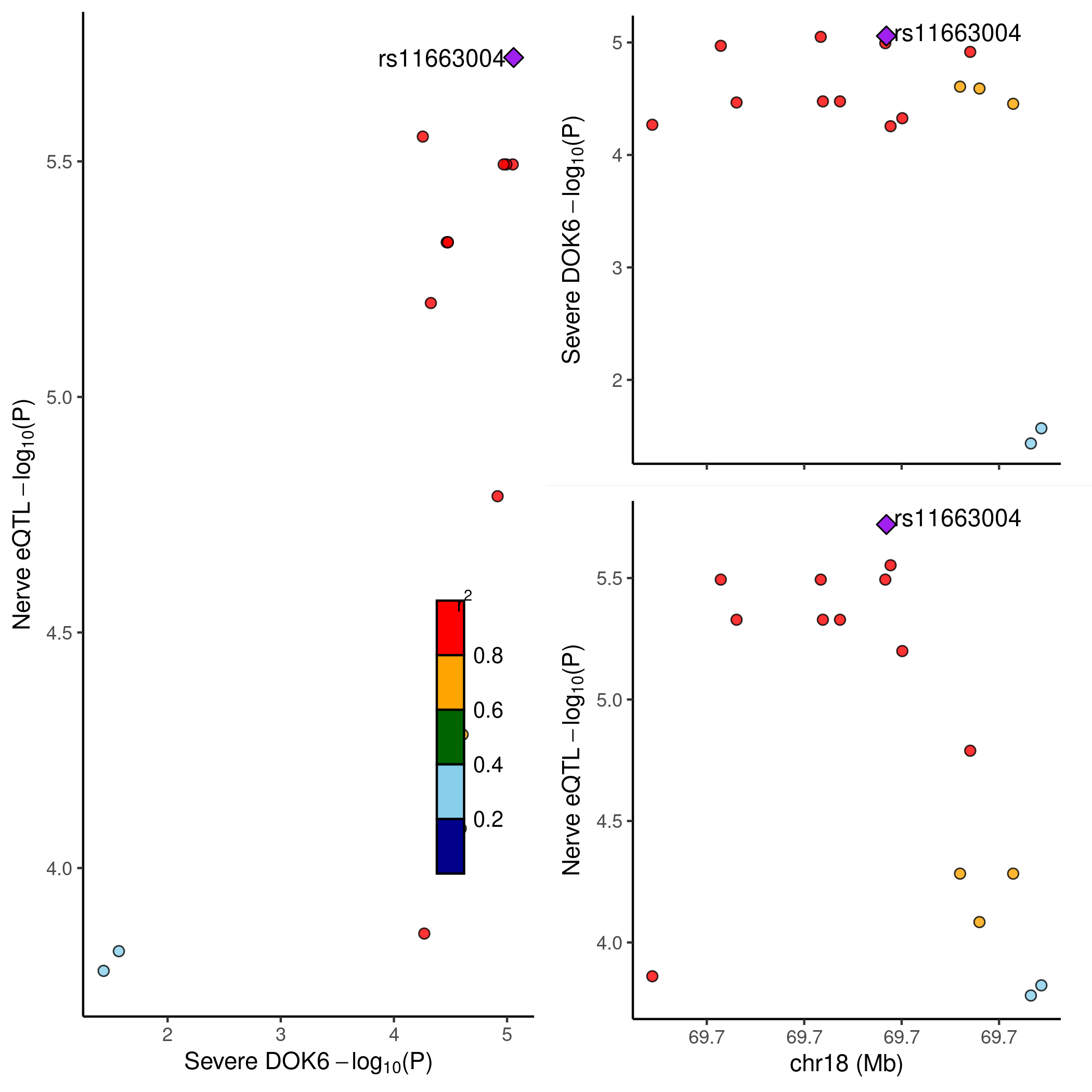


Figure S9

Locuscompare Plot for Severe Pain DOK6 and Nerve Tibial eQTL. Top right: regional scatter plot for GWAS. Bottom right: regional scatter plot for eQTL. Left: joint distribution of p-values from GWAS and eQTL. Color represents LD with lead SNP.
